# Pangenome-wide analysis of cyclic nucleotide-gated channel (CNGC) gene family in citrus *Spp*. Revealed their intraspecies diversity and potential roles in abiotic stress tolerance

**DOI:** 10.3389/fgene.2022.1034921

**Published:** 2022-10-11

**Authors:** Komal Zia, Muhammad Junaid Rao, Muhammad Sadaqat, Farrukh Azeem, Kinza Fatima, Muhammad Tahir ul Qamar, Abdulrahman Alshammari, Metab Alharbi

**Affiliations:** ^1^ Integrative Omics and Molecular Modeling Laboratory, Department of Bioinformatics and Biotechnology, Government College University Faisalabad (GCUF), Faisalabad, Pakistan; ^2^ State Key Laboratory for Conservation and Utilization of Subtropical Agro-Bioresources, Guangxi Key Laboratory of Sugarcane Biology, College of Agriculture, Guangxi University, Nanning, China; ^3^ Department of Botany and Plant Sciences, University of California Riverside (UCR), Riverside, CA, United States; ^4^ Department of Pharmacology and Toxicology, College of Pharmacy, King Saud University, Riyadh, Saudi Arabia

**Keywords:** CNGC, citrus, pan-genomics, drought stress, genome-wide analysis, molecular modeling

## Abstract

Cyclic nucleotide-gated channels (CNGC) gene family has been found to be involved in physiological processes including signaling pathways, environmental stresses, plant growth, and development. This gene family of non-selective cation channels is known to regulate the uptake of calcium and is reported in several plant species. The pangenome-wide studies enable researchers to understand the genetic diversity comprehensively; as a comparative analysis of multiple plant species or member of a species at once helps to better understand the evolutionary relationships and diversity present among them. In the current study, pangenome-wide analysis of the CNGC gene family has been performed on five Citrus species*.* As a result, a total of 32 genes in *Citrus sinensis*, 27 genes in *Citrus recticulata*, 30 genes in *Citrus grandis*, 31 genes in *Atalantia buxfolia,* and 30 genes in *Poncirus trifoliata* were identified. In addition, two unique genes *CNGC13* and *CNGC14* were identified, which may have potential roles. All the identified CNGC genes were unevenly distributed on 9 chromosomes except *P. trifoliata* had genes distributed on 7 chromosomes and were classified into four major groups and two sub-groups namely I, II, III, IV-A, and IV-B. Cyclic nucleotide binding (CNB) motif, calmodulin-binding motif (CaMB), and motif for IQ-domain were conserved in Citrus *Spp.* Intron exon structures of citrus species were not exactly as same as the gene structures of *Arabidopsis*. The majority of cis-regulatory elements (CREs) were light responsive and others include growth, development, and stress-related indicating potential roles of the CNGC gene family in these functions. Both segmental and tandem duplication were involved in the expansion of the CNGC gene family in Citrus *Spp.* The miRNAs are involved in the response of CsCNGC genes towards drought stress along with having regulatory association in the expression of these genes. Protein- Protein interaction (PPI) analysis also showed the interaction of CNGC proteins with other CNGCs which suggested their potential role in pathways regulating different biological processes. GO enrichment revealed that CNGC genes were involved in the transport of ions across membranes. Furthermore, tissue-specific expression patterns of leaves sample of *C. sinensis* were studied under drought stress. Out of 32 genes of *C. sinensis* 3 genes i.e., *CsCNGC1.4*, *CsCNGC2.1,* and *CsCNGC4.2* were highly up-regulated, and only *CsCNGC4.6* was highly down-regulated. The qRT-PCR analysis also showed that CNGC genes were highly expressed after treatment with drought stress, while gene expression was lower under controlled conditions. This work includes findings based on multiple genomes instead of one, therefore, this will provide more genomic information rather than single genome-based studies. These findings will serve as a basis for further functional insights into the CNGC gene family.

## 1 Introduction

Calcium is an important macronutrient for plant growth and development and is involved in signaling pathways as a secondary messenger. It also plays a key role in the defense mechanism of plants against abiotic stress (Lecourieux et al., 2006; Kudla et al., 2018). Calcium sensor proteins belong to three main families including calmodulin (CaM) and calmodulin-like proteins (CMLs) (Yang and Poovaiah, 2003; Bender and Snedden, 2013), calcineurin-B-like proteins (CBLs) (Luan, 2009), calcium dependent protein kinases (CPKs) and calcium and calmodulin dependent protein kinase (CCaMK) (Cheng et al., 2002; Wang et al., 2015). Calcium binding to these calcium sensors induces a conformational change that triggers either a particular target protein or directly stimulates kinase activity by taking into account CPKs (Ranty et al., 2016). In contrast, several families of ion channels regulate the uptake of calcium including Cyclic nucleotide-gated channels (CNGCs), two pore channel 1 (TCP1), ionotropic glutamate receptors, and several other channels (Demidchik et al., 2018).

CNGCs belong to the nonselective cation channels that are found in both animals and plants. Plant CNGCs was first discovered in 1998 while scanning calmodulin-conjugated transporters (HvCBT1) in barley (Mäser et al., 2001). CNGCs are ligand-gated channels that are calcium permeable and involved in the interaction of cyclic nucleotides and calcium dependent signaling pathways (Talke et al., 2003). CNGCs are calcium sensors in eukaryotes while calcium is important for plant growth, development, light signaling, drought and salt stress, and pathogen tolerance (Ranty et al., 2016). CNGCs get activated by the binding of cyclic nucleotides (cNMP) and their activity gets inhibited by Ca^2+^/CaM binding (Trudeau and Zagotta, 2002). Calcium is very helpful in regulating plant growth under stress conditions. There are 6 TM domains (S1-S6) and a pore region in CNGCs, fifth and sixth domains along with the Cyclic nucleotide-binding domain (CNBD) and CaM binding domains are present at C-terminal. CNBD comprises a phosphate binding cassette (PBC) and a hinge region (Duszyn et al., 2019). The PBC binds to phosphate and sugar moieties of cyclic nucleotide binding (CNB) ligand and the hinge region contributes to the efficacy of ligand binding and selectivity (Li et al., 2019). CNGCs are also involved in plants responses to various abiotic and biotic stress conditions. (Jha et al., 2016).

CNGC gene family has been reported in *Arabidopsis thaliana* (Mäser et al., 2001), *Brassica oleracea* (Kakar et al., 2017), *Zea mays* (Hao and Qiao, 2018), *Ziziphus jujube Mill.* (Wang et al., 2020), *Nicotiana tobacum L.* (Nawaz et al., 2018), *Triticum aestivum L.* (Guo et al., 2018)*, Oryza sativa* (Nawaz et al., 2014)*, Brassica rapa* (Li et al., 2019)*, Pyrus bretschneideri Rehd* (Chen et al., 2015). and *Solanum lycopersicum* (Saand et al., 2015). On the basis of the phylogenetic classification in the aforementioned plants, this gene family is classified into four major groups and the fourth group is further divided into two sub-groups namely as; I, II, III, IV-A, IV-B. A single reference genome is not enough to capture diversity present among the members of a species (Golicz et al., 2016). Thus, it brings a bias to study gene family members in plants solely based on a single genome. Therefore, it is suggested to conduct pangenome-wide analysis for gene family characterization (Tahir Ul Qamar et al., 2019). The first ever concept regarding pangenome was introduced when the pangenome of *Streptococcus agalacitae* was developed (Tettelin et al., 2005). Pangenome of a species comprises core genes that are present in all members, accessory genes that are present in few but not in all members, and unique genes that are present only in specific members (Tahir ul Qamar et al., 2020; Ismail et al., 2022; Zanini et al., 2022).

Citrus is an economically important fruit crop as it is widely used both as a fruit and as a juice (Liu et al., 2019). It is perennial crop and mostly cultivated in China, Brazil, India, United States, Mexico, Spain, and Italy (Liu et al., 2012). Citrinae is a large group of citrus fruit trees that belong to the subfamily Aurantioideae and the family Rutaceae. Based on botanical features Citrinae is categorized into three types i.e., primitive citrus, near citrus, and true citrus (Wang et al., 2017). The well-known Citrus varities include; *Atlantia buxfolia* (Chinese box orange), *Citrus sinensis* (sweet orange), *Citrus grandis* (pummelo), *Citrus recticulata* (mandarin), *Citrus limon* (lemon), *Citrus paradisi* (grapefruit) and *Poncirus trifoliata* (Trifoliate orange) (Liu et al., 2019). Citrus varities widely influenced by drought stress as the productivity, growth, and yield of citrus get reduced after facing drought stress (Osakabe et al., 2014). However, few drought resistant varities are also reported which can withstand against this stress, including navel orange and trifoliate orange (Bhusal et al., 2002; Koshita and Takahara, 2004; Pingping et al., 2017).

In present study, *C. sinensis, C. recticulata, C. grandis, A. buxfolia,* and *P. trifoliata* were selected for pangenome-wide analysis of CNGCs gene family, as they have good quality assembled genomes and their annotations are available at chromosome level. The quality of genome assembly or sequencing directly affects the quality of results (Vaattovaara et al., 2019), therefore, the aforementioned species were preferred to reduce the biasness. CNGCs gene family has been studied in several plant species at single genome-wide level (Mäser et al., 2001; Nawaz et al., 2014, 2018; Chen et al., 2015; Saand et al., 2015; Kakar et al., 2017; Guo et al., 2018; Hao and Qiao, 2018; Li et al., 2019; Wang et al., 2020), but no pan-genome-wide analysis has been performed before. Therefore, current study aims to provide a comprehensive pangenome-wide representation of CNGCs gene family in citrus species, which will serve as the foundation for future gene family researches.

## 2 Materials and methods

### 2.1 Identification of cyclic nucleotide-gated channel family genes in *C. sinensis, C. recticulata, C. grandis, A. buxfolia,* and *P. trifoliata*

20 CNGC protein sequences of *A. thaliana* taken from TAIR database (https://www.arabidopsis.org/) (Rhee et al., 2003) were used as query and BLASTp search was performed on Citrus pan-genome to breeding database (CPBD; https://citrus.hzau.edu.cn/) (Liu et al., 2022) against *C. sinensis* v2.0, *A. buxfolia* v2.0, *P. trifoliata* v1.0, *C. recticulata* v2.0, and *Citrus grandis (L.) Osbeck.* cv. *Wanbaiyou* v1.0. The resulting BLAST hits were manually processed to remove duplicates and isoforms and the final hits were used for further analyses.

To check the presence of specific domains, databases including SMART (https://smart.embl-heidelberg.de/) (Schultz et al., 2000), CDD (https://pfam.xfam.org/) (Marchler-bauer et al., 2011), and HMMER (https://www.ebi.ac.uk/Tools/hmmer/search/hmmscan) (Potter et al., 2018) were used. This eliminated those sequences that didn’t have specific conserved domains required for CNGC protein function. Domain architecture was constructed using the HMMER database. Molecular weight (MW), Theoretical isoelectric point (PI), Instability index (II), Aliphatic index (AI), and Grand average of hydropathy (GRAVY) were determined by using the web-based tool ProtParam available at the EXPASY server (https://web.expasy.org/protparam) (Gasteiger et al., 2003). Subcellular localization was determined using CELLO version 2.5 (https://cello.life.nctu.edu.tw/) (Yu et al., 2006).

### 2.2 Multiple sequence alignment and phylogenetic analysis

To comprehend the phylogenetic relationships of identified *CNGCs*, multiple sequence alignment of identified CNGC protein sequences of *C. sinensis*, *A. buxfolia*, *C. recticulata*, *C. grandis*, *P. trifoliata* along with already reported protein sequences of *O. sativa* (Nawaz et al., 2014), *Z. jujuba* (Wang et al., 2020)*, Z. mays* (Hao and Qiao, 2018), *A. thaliana* (Köhler and Neuhaus, 2000) and *P. bretschneideri* (Chen et al., 2015) was done using ClustalW program and a phylogenetic tree was constructed by using online server IQ-tree (https://iqtree.cibiv.univie.ac.at/) (Nguyen et al., 2015) with Maximum Likelihood (ML) method and 1,000 replicates while other parameters were set to their default values. The tree was visualized and edited using the online server iTOL (https://itol.embl.de/) (Letunic and Bork, 2021).

### 2.3 Chromosomal location, gene structure, and conserved motif analysis

The chromosomal location, start and end sites of *C. sinensis*, *A. buxfolia*, *C. recticulata*, *C. grandis,* and *P. trifoliata* were retrieved from the CPBD database and a genetic linkage was constructed by using TBtools (Chen et al., 2020). The gene and CDS sequences of *C. sinensis*, *A. buxfolia*, *C. recticulata*, *C. grandis,* and *P. trifoliata* were retrieved from the sequence fetch option at the CPBD database (https://citrus.hzau.edu.cn/) (Liu et al., 2022). The GSDS v2.0 (https://gsds.gao-lab.org/) (Hu et al., 2015) was used for the visualization of gene structures of CsCNGCs, AbuCNGCs, CreCNGCs, CgCNGCs, and PtCNGCs. Conserved motifs were identified by using MEME (Multiple EM for Motif Elicitation) suite 5.4.1 (https://meme-suite.org/meme/db/motifs) (Bailey et al., 2009). All parameters were set to their default values except the number of motifs that were set to 10.

### 2.4 Gene duplication and promoter analysis

The location of *CNGC* genes in *C. sinensis, C. recticulata, C. grandis, A. buxfolia,* and *P. trifoliata* was retrieved from the CPBD database (https://citrus.hzau.edu.cn/) (Liu et al., 2022). All genes possessing ≥70% sequence identity were considered duplicated genes (Hu et al., 2021). DnaSP v6.0 (Librado and Rozas, 2009) offline tool was used to calculate the rate of Non-synonymous (Ka) and synonymous substitutions (Ks) of duplicated gene pairs. To calculate the selection pressure that assisted in the evolution of the *CNGC* gene family Ka/Ks ratio was used. The formula for calculating duplication time was the following: *T* = *Ks*/2*x* (where *x* represents substitutions per synonymous site per year and is equal to 6.56 × 10^−9^ for dicots) (He et al., 2016). The cis-elements in 2000bp coding regions of *CsCNGCs*, *CreCNGCs*, *CgCNGCs*, *AbuCNGCs,* and *PtCNGCs* were retrieved from the Citrus pan-genome to breeding database (CPBD, https://www.citrus.hzau.edu.cn/) (Liu et al., 2022). While the types, numbers, and functions of these cis-elements were analyzed by using PlantCare web-based tool (https://bioinformatics.psb.ugent.be/webtools/plantcare/html/) (Lescot et al., 2002).

### 2.5 Putative miRNA target prediction, protein-protein interaction network, and gene ontology analysis of citrus *Spp.*

Plant microRNA Encyclopedia (PmiREN; https://pmiren.com) database was utilized to acquire mature miRNA sequences of *C. sinensis.* For putative miRNA target prediction CDS sequences of the potential target, *CsCNGCs* were utilized and were submitted at the psRNATarget server (https://www.zhaolab.org/psRNATarget/home) (Dai et al., 2018) along with the respective mature miRNA sequences of *C. sinensis* with default considerations. The regulatory association between target *CsCNGCs* and predicted miRNAs was visualized using Cytoscape software (Shannon et al., 1971). The interaction among members of the CNGC protein family and other proteins from the citrus plant was predicted using the STRING database (https://string-db.org/). 32 CsCNGC protein sequences were uploaded to the STRING database with ‘*Citrus sinensis*’ being selected as reference species. The level of connection used was sixth and other parameters were kept by default. PPI network was visualized and edited using Cytoscape software (Shannon et al., 1971). Citrus Pan-genome2breeding database (CPBD; http://citrus.hzau.edu.cn/) (Liu et al., 2022) was utilized to analyze gene ontology (GO) enrichment of Citrus *Spp.* using the gene IDs of *CNGC* genes.

### 2.6 Expression profiling of *C. sinensis* under drought stress

To demonstrate the expression of *C. sinensis* under abiotic stress (drought) in leaves, RNA-seq data was downloaded from the NCBI-SRA database (https://www.ncbi.nlm.nih.gov/sra) (BioProject: PRJNA792482). Reference genome and GFF3 files were downloaded from the Citrus pan-genome to breeding database (CPBD, https://citrus.hzau.edu.cn/) (Liu et al., 2022). To check the quality of paired-end data (in FASTQ format) FASTQC was utilized and Trimmomatic was used for trimming and improving the quality of reads. Then HISAT2 was used for the alignment of reads to the C. *sinensis v2.0* genome. To normalize gene expression in terms of Fragments per kilobase of transcripts per million mapped reads (FPKM) Cufflinks were used. The heatmap was constructed using pheatmap function of R-language (Ihaka and Gentleman, 1996).

### 2.7 Drought stress treatment, ribonucleic acid isolation, and quantitative real-time reverse transcription–polymerase chain

Citrus plants were grown under controlled environmental conditions in a growth chamber (having 60 ± 3% humidity, 27 ± 2°C temperature, and 5000 LUX light intensity) with recommended fertilizer and water treatment. Four months old citrus plants were subjected to drought stress and leaves were collected and 0, 10, and 20 days of drought stress. Control and drought-stressed leaves were harvested for RNA extraction. Zomanbio (Cat no. ZP401-2) total RNA-pure reagent (Lot#200F12F) was used to extract total RNA and the complementary DNA (cDNA) was synthesized by using Zomanbio (M-MLV, ZR102-3) reverse transcriptase kit (Beijing, ZOMAN Biotechnology Co., Ltd.) according to the manufacturer instructions. For quantitative real-time polymerase chain reaction (qRT-PCR) ChamQ universal master mix SYBR (Vazyme, Q711-02) and LongGene (Model: q2000b) fluorescence quantitative PCR instrument (Langji Scientific instrument Co., Ltd.; Hangzhou, China) were used whereas citrus actin gene was used as an internal reference. 2^-(ΔΔCt) method was applied to analyze the qRT-PCR expression data in Excel (Microsoft Corp., Redmond, WA, United States). Statistix 8.1 (Tallahassee Florida, United States) statistical software was used for analyzing all qRT-PCR data and the Excel program was used for graphs. The qPCR primer information is characterized (Supplementary Table S1).

### 2.8 3D Structure prediction of cyclic nucleotide-gated channels in citrus spp.

Three-dimensional (3D) structures of 13 CNGC proteins were predicted, including 9 proteins from *C. sinensis*, one from *A. buxfolia,* and two from *P. trifoliata.* Among these 13 proteins, 3D structures of 12 CNGC proteins were predicted by using Alphafold2 (https://colab.research.google.com/github/sokrypton/ColabFold/blob/main/AlphaFold2.ipynb) (Jumper et al., 2021) Whereas, the 3D structure of CsCNGC1.4 was predicted by using trRosetta (https://yanglab.nankai.edu.cn/trRosetta/) (Du et al., 2021) due to its length i.e., 1427aa. Protein structures were visualized by using Pymol (Yuan et al., 2017). For validation of these predicted structures SAVES server (https://saves.mbi.ucla.edu) was used.

## 3 Results

### 3.1 Identification of cyclic nucleotide-gated channel genes in *C. sinensis, C. recticulata, C. grandis, A. buxfolia,* and *P. trifoliata*

**FIGURE 1:**
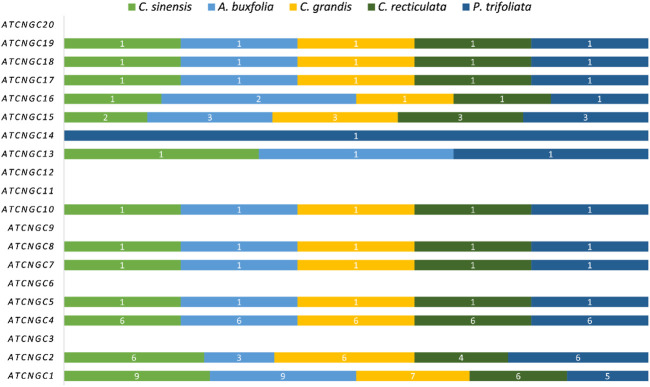
Bar Plot showing homologs of *Arabidopsis* CNGCs present in five Citrus species. Each species is having a variable number of members.

A total of 32 putative genes in *C. sinensis*, 27 genes in *C. recticulata*, 30 genes in *C. grandis*, 31 genes in *A. buxfolia,* and 30 in *P. trifoliata* were identified. The identified *CNGC* genes were named based on their phylogenetic relationships with *CNGCs* in *Arabidopsis*. Figure 1 is showing the homologs of *Arabidopsis CNGC* genes present in five species under study. Most of the identified members of *Arabidopsis CNGCs* are present in five species under study except for *AtCNGC3*, *AtCNGC6*, *AtCNGC9*, *AtCNGC11*, *AtCNGC12,* and *AtCNGC20*. All other members have a variable number of homologs present in five Citrus species. Further, two unique genes were identified: *CNGC13* and *CNGC14*. *CNGC13* is present in three plant species including *C. sinensis*, *A. buxfolia,* and *P. trifoliata* while absent in *C. grandis* and *C. reticulata*. *CNGC14* is present in only one plant species, *P. trifoliata* while being absent in the other four species.

**FIGURE 2:**
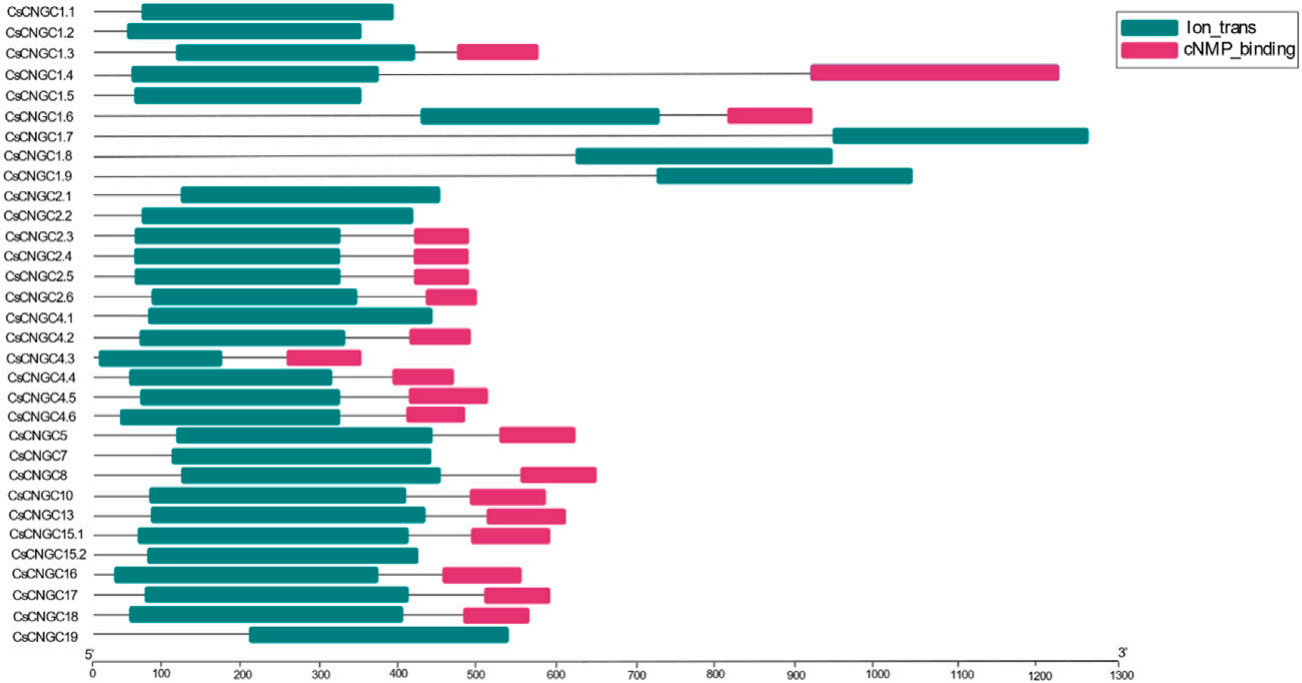
Domain architecture of *C. sinensis* cyclic nucleotide-gated channels (CsCNGCs) proteins.

Conserved domains that were predicted in *C. sinensis*, *A. buxfolia*, *C. recticulata*, *C. grandis,* and *P. trifoliata* include Cyclic Nucleotide Binding Domain (CNBD or cNMP), Ion trans (IT), Cap family effector domain (CAP_ED) and other ion trans domains (Supplementary Table S2). Ion trans and cNMP binding domains were the most conserved among all. Domain architecture was constructed according to the prediction results of the HMMER database. cNMP binding domain was not present in CsCNGC1.1, CsCNGC1.2, CsCNGC1.5, CsCNGC1.7, CsCNGC1.8, CsCNGC1.9, CsCNGC2.1, CsCNGC2.2, CsCNGC4.1, CsCNGC7, CsCNGC15.2 and CsCNGC19 according to prediction results of HMMER database but SMART database prediction confirms the presence of cNMP binding domain in these proteins. The domain architecture of *C. sinensis* is given in (Figure 2).

Ion trans domain was absent in CreCNGC1.2, and CreCNGC1.6 according to HMMER database prediction. While CDD prediction confirms the presence of the Ion trans domain in CreCNGC1.6. Results of the HMMER database demonstrate the absence of the cNMP binding domain in CreCNGC1.1, CreCNGC1.3, CreCNGC1.4, CreCNGC2.1, CreCNGC4.1, CreCNGC7, CreCNGC15.1, CreCNGC15.3, and CreCNGC19 according to prediction. Domains predicted by the SMART database indicated the presence of the cNMP binding domain in these proteins (Supplementary Figure S1).

The following proteins of *C. grandis* CgCNGC1.1, CgCNGC1.2, CgCNGC1.3, CgCNGC1.4, CgCNGC1.5, CgCNGC1.6, CgCNGC2.1, CgCNGC2.2, CgCNGC4.1, CgCNGC7, CgCNGC15.1, CgCNGC15.3, and CgCNGC19 didn’t have cNMP binding domain as per HMMER database prediction. Whereas, the cNMP binding domain was predicted to be present in all these proteins except CgCNGC1.3 as supported by SMART prediction (Supplementary Figure S2).

AbuCNGC1.1, AbuCNGC1.3, AbuCNGC1.5, AbuCNGC1.7, AbuCNGC1.8, AbuCNGC1.9, AbuCNGC2.1, AbuCNGC4.1, AbuCNGC7, AbuCNGC15.1, AbuCNGC15.3, and AbuCNGC19 are those aforementioned proteins of *A. buxfolia* that have cNMP binding domain absent in them according to the prediction of HMMER database. But taking into account the domains predicted in these proteins by the SMART database the cNMP binding domain was present in all of them. Prediction results of the HMMER, SMART, and CDD database demonstrate the absence of the Ion trans domain in AbuCNGC1.4 (Supplementary Figure S3).

PtCNGC proteins that have cNMP binding domain absent in them include PtCNGC1.1, PtCNGC1.3, PtCNGC1.4, PtCNGC1.5, PtCNGC2.1, PtCNGC2.2, PtCNGC2.3, PtCNGC2.4, PtCNGC4.1, PtCNGC7, PtCNGC14, PtCNGC15.1, PtCNGC15.3 and PtCNGC19 as predicted by HMMER database. But the cNMP binding domain was absent only in PtCNGC14 and present in all the other aforementioned PtCNGCs (Supplementary Figure S4). Details of CNGCs reported in other plants are shown in Table 1.

**TABLE 1:** Summary of CNGCs reported in other plants.

Plant name	Cotyledon	Group I	Group II	Group III	Group IV-A	Group IV-B	Total	References
*Arabidopsis thaliana*	Dicot	6	5	5	2	2	20	Mäser et al. (2001)
*Brassica rapa*	Dicot	7	5	6	9	3	30	Li et al. (2019)
*Brassica oleracea*	Dicot	3	5	6	3	9	26	Kakar et al. (2017)
*Zea mays*	monocot	3	2	3	1	3	12	Hao and Qiao, (2018)
*Pyrus bretschneideri Rehd.*	Dicot	7	2	5	2	5	21	Chen et al. (2015)
*Oryza sativa*	monocot	3	3	5	2	3	16	Nawaz et al. (2014)
*Ziziphus jujuba Mill.*	Dicot	3	2	6	1	3	15	Wang et al. (2020)
*Triticum aestivum L.*	monocot	4	12	19	4	8	47	Guo et al. (2018)
*Nicotiana tabacum L.*	Dicot	7	6	12	3	7	35	Nawaz et al. (2018)
*Solanum lycopersicum L.*	Dicot	6	3	5	1	3	18	Saand et al. (2015)

### 3.2 Physiochemical properties and subcellular localization analysis of cyclic nucleotide-gated channels in citrus *Spp.*

The detailed physio-chemical properties of 150 CNGC proteins of five Citrus *Spp.* are shown in (Table 2). *C. sinensis* had protein length ranging from 492–1553aa, molecular weight (MW) ranging from 56.13–177.77 (KDa), and Isoelectric point (PI) ranging from 6.38–9.52, Instability index (II) was above 40 for 25 proteins of *C. sinensis*, indicating that most of the proteins were unstable. GRAVY values of 29 proteins of *C. sinensis* were negative indicating that the majority of proteins were hydrophilic. Results of subcellular localization suggested that all putative CsCNGC proteins were present in the plasma membrane.

**TABLE 2:** Physiochemical properties of Citrus *Spp.*

Gene Name	Protein id	Group	TM domains	Chr	Start	End	Strand	Protein length (AA)	Molecular weight (MW)	Isoelectric point (PI)	Instability index (II)	Aliphatic index (AI)	Grand average of hydropathy (GRAVY)	Subcellular localization
*C. sinensis* CNGCs															
CsCNGC1.1	Cs9g_pb020800.1	I	6	9	24,382,883	24,387,758	+	927	106,182.81	9.47	49.27-unst	92.39	-0.115	Plasma membrane	
CsCNGC1.2	Cs9g_pb020720.2	I	5	9	24,280,566	24,287,342	+	649	74,468.57	9.07	41.33-unst	90.59	-0.044	Plasma membrane	
CsCNGC1.3	Cs2g_pb014570.1	I	5	2	12,798,466	12,807,926	-	668	77,016.05	9.23	50.97-unst	92.67	-0.052	Plasma membrane	
CsCNGC1.4	Cs9g_pb020710.1	I	11	9	24,272,155	24,279,547	-	1,427	164,185.99	9.01	48.08-unst	89.68	-0.222	Plasma membrane	
CsCNGC1.5	Cs9g_pb020550.1	I	4	9	2,4,081,889	24,084,603	-	572	65,811.13	8.57	40.46-unst	98.42	-0.016	Plasma membrane	
CsCNGC1.6	Cs9g_pb020690.1	I	6	9	24,259,820	24,265,542	-	958	109,207.77	8.47	43.41-unst	89.23	-0.18	Plasma membrane	
CsCNGC1.7	Cs9g_pb020610.1	I	12	9	24,129,588	24,139,417	-	1,553	177,772.54	8.3	42.08-unst	95.86	-0.04	Plasma membrane	
CsCNGC1.8	Cs9g_pb020780.1	I	12	9	24,345,495	24,353,666	+	1,181	136,124.69	8.62	43.7-unst	93.65	0.018	Plasma membrane	
CsCNGC1.9	Cs9g_pb020790.1	I	11	9	24,357,999	24,370,147	+	1,290	147,302.3	8.7	40.01-unst	101.17	0.09	Plasma membrane	
CsCNGC2.1	Cs6g_pb020330.1	IV-B	7	6	21,816,537	21,821,804	+	714	81,992.71	9.51	48.62-unst	93.24	-0.024	Plasma membrane	
CsCNGC2.2	Cs6g_pb020320.1	IV-B	6	6	21,803,582	21,807,522	-	668	76,724.79	9.44	51.02-unst	100.24	0.04	Plasma membrane	
CsCNGC2.3	Cs9g_pb014190.4	IV-B	5	9	17,045,621	17,063,515	+	839	95,658.31	6.72	38.76	97.02	-0.081	Plasma membrane	
CsCNGC2.4	Cs3g_pb003680.1	IV-B	5	3	9,615,973	9,627,834	+	817	93,479.69	6.62	35.54	97	-0.111	Plasma membrane	
CsCNGC2.5	Cs8g_pb010740.1	IV-B	5	8	11,455,619	11,467,550	-	817	93,333.42	6.72	35.97	96.04	-0.131	Plasma membrane	
CsCNGC2.6	Cs1g_pb001090.1	IV-B	5	1	3,825,286	3,830,902	-	822	94,303.4	6.42	39.7	96.55	-0.18	Plasma membrane	
CsCNGC4.1	CsUn_pb001730.1	IV-B	7	un	2,087,324	2,092,767	-	696	80,863.76	8.8	55.85-unst	88.92	-0.174	Plasma membrane	
CsCNGC4.2	Cs3g_pb003330.1	IV-B	5	3	5,449,938	5,460,243	-	834	95,075.26	7.35	34	99.74	-0.08	Plasma membrane	
CsCNGC4.3	Cs4g_pb023890.1	IV-B	3	4	24,723,554	24,729,965	+	492	56,135.04	7.33	36.1	100.45	-0.08	Plasma membrane	
CsCNGC4.4	Cs2g_pb007660.1	IV-B	6	2	2,423,659	2,427,730	-	785	89,767.74	6.38	45.13-unst	89.66	-0.202	Plasma membrane	
CsCNGC4.5	Cs4g_pb023340.1	IV-B	5	4	25,225,304	25,229,876	-	888	99,688.53	7.64	44.69-unst	97.72	-0.133	Plasma membrane	
CsCNGC4.6	Cs4g_pb019870.1	IV-B	5	4	21,495,102	21,501,116	-	884	99,608.21	6.68	38.99	96.47	-0.135	Plasma membrane	
CsCNGC5	Cs4g_pb000740.1	II	5	4	952,948	960,923	-	734	84,009.72	9.04	49.85-unst	87.56	-0.171	Plasma membrane	
CsCNGC7	Cs1g_pb010020.2	II	5	1	15,065,043	15,071,550	+	747	85,912.18	9.25	46.4-unst	93.82	-0.137	Plasma membrane	
CsCNGC8	Cs1g_pb010030.1	II	5	1	15,075,187	15,080,475	+	749	86,209.43	9.22	50.26-unst	86.28	-0.189	Plasma membrane	
CsCNGC10	Cs9g_pb020770.1	I	5	9	24,338,616	24,343,695	+	710	81,613.47	9.14	46.55-unst	88.84	-0.136	Plasma membrane	
CsCNGC13	Cs5g_pb002630.1	I	5	5	1,720,153	1,724,660	+	723	83,314.54	9.17	51.42-unst	88.2	-0.184	Plasma membrane	
CsCNGC15.1	Cs8g_pb020390.1	III	5	8	20,533,061	20,537,081	+	698	80,196.86	9.3	52.12-unst	92.18	-0.147	Plasma membrane	
CsCNGC15.2	Cs6g_pb003650.1	III	6	6	7,204,408	7,208,020	+	711	81,667.77	9.3	52.11-unst	91.04	-0.102	Plasma membrane	
CsCNGC16	Cs5g_pb011010.1	III	6	5	5,013,563	5,016,560	+	691	79,200.38	8.1	51.35-unst	95.55	-0.043	Plasma membrane	
CsCNGC17	Cs4g_pb001750.1	III	5	4	1,625,519	1,631,583	+	723	83,201.18	9.38	40.68-unst	91.43	-0.187	Plasma membrane	
CsCNGC18	Cs2g_pb028120.1	III	7	2	3,1,331,822	31,335,320	-	732	83,366.68	8.38	44.03-unst	91.53	-0.053	Plasma membrane	
CsCNGC19	Cs5g_pb020580.2	IV-A	6	5	22,133,938	22,155,313	-	775	89,042.58	9.52	45.27-unst	86.69	-0.222	Plasma membrane	
C. recticulata CNGCs															
CreCNGC1.1	Cre9g_024,150.1	I	5	9	30,685,880	30,690,506	-	961	109,420.49	9.44	48.06-unst	93.09	-0.101	Plasma membrane	
CreCNGC1.2	Cre2g_019,820.1	I	1	2	16,922,647	16,925,029	-	371	43,157.28	8.42	55.6-unst	81.19	-0.359	Plasma membrane	
CreCNGC1.3	Cre9g_024,250.1	I	5	9	30,796,869	3,0,801,729	+	958	109,413.81	8.96	48.74-unst	85.85	-0.339	Plasma membrane, Nuclear	
CreCNGC1.4	Cre9g_024,430.1	I	4	9	30,990,029	30,992,744	+	572	65,837.21	8.57	40.31-unst	99.11	-0.006	Plasma membrane	
CreCNGC1.5	Cre9g_024,260.1	I	6	9	30,808,308	30,814,126	+	962	109,643.04	8.62	41.49-unst	87.34	87.34	Plasma membrane	
CreCNGC1.6	Cre9g_024,380.1	I	12	9	30,935,435	30,944,980	+	1,513	173,290.51	8.49	42.46-unst	96.34	-0.029	Plasma membrane	
CreCNGC2.1	Cre6g_023,700.1	IV-B	6	6	25,763,432	25,767,287	-	671	77,122.25	9.38	51.84-unst	99.21	0.037	Plasma membrane	
CreCNGC2.2	Cre9g_017,070.1	IV-B	3	9	23,979,122	23,990,027	+	583	66,299.41	6.52	35.75	99.64	-0.094	Plasma membrane	
CreCNGC2.3	Cre9g_014,190.1	IV-B	5	9	16,533,233	16,544,587	-	816	93,352.54	6.9	33.85	96.39	-0.126	Plasma membrane	
CreCNGC2.4	Cre1g_003,650.1	IV-B	5	1	3,866,987	3,872,463	-	822	94,327.41	6.33	39.86	97.03	-0.181	Plasma membrane	
CreCNGC4.1	Cre4g_013,910.1	IV-B	7	4	19,140,542	19,146,068	+	696	80,893.79	8.8	55.85-unst	88.78	-0.178	Plasma membrane	
CreCNGC4.2	Cre3g_025,930.2	IV-B	7	3	29,055,761	29,066,087	+	869	99,262.3	7.14	33.61	100.99	-0.04	Plasma membrane	
CreCNGC4.3	Cre4g_003,630.1	IV-B	6	4	2,657,893	2,664,366	-	633	72,434.98	8.78	36.21	101.49	0.002	Plasma membrane	
CreCNGC4.4	Cre2g_002,210.1	IV-B	5	2	1,245,194	1,249,035	-	785	89,717.54	6.53	44.43-unst	88.79	-0.226	Plasma membrane	
CreCNGC4.5	Cre4g_002,970.1	IV-B	5	4	2,152,171	2,156,534	+	888	99,743.65	7.92	44.94-unst	97.84	-0.137	Plasma membrane	
CreCNGC4.6	Cre4g_006,610.1	IV-B	5	4	5,039,735	5,045,661	+	884	99,723.29	6.79	39.14	96.02	-0.143	Plasma membrane	
CreCNGC5	Cre4g_026,070.1	II	5	4	28,785,823	28,793,750	+	735	84,068.75	9.04	50.16-unst	87.18	-0.175	Plasma membrane	
CreCNGC7	Cre1g_009,420.1	II	5	1	14,655,919	14,661,193	+	747	85,925.18	9.25	45.18-unst	93.43	-0.143	Plasma membrane	
CreCNGC8	Cre1g_009,430.1	II	2	1	14,666,579	14,670,161	+	534	61,818.9	8.86	50.19-unst	82.87	-0.298	Plasma membrane	
CreCNGC10	Cre9g_024,180.1	I	5	9	30,730,436	30,735,418	-	710	81,613.47	9.14	46.55-unst	88.84	-0.136	Plasma membrane	
CreCNGC15.1	Cre8g_016,390.1	III	7	8	1,5,741,807	15,745,297	-	729	83,420.51	9.13	49.51-unst	92.25	-0.121	Plasma membrane	
CreCNGC15.2	Cre8g_016,380.1	III	5	8	15,732,273	15,735,541	-	698	80,196.86	9.3	52.12-unst	92.18	-0.147	Plasma membrane	
CreCNGC15.3	Cre6g_004,960.1	III	6	6	10,274,134	10,277,745	+	711	81,722.85	9.33	52.22-unst	91.04	-0.108	Plasma membrane	
CreCNGC16	Cre5g_004,550.1	III	4	5	3,074,499	3,077,145	+	604	69,195.59	7.93	50.73-unst	93.33	-0.111	Plasma membrane	
CreCNGC17	Cre4g_025,000.1	III	5	4	28,114,436	28,119,138	-	723	83,242.23	9.34	41.19-unst	91.43	-0.188	Plasma membrane	
CreCNGC18	Cre2g_027,400.1	III	7	2	27,779,686	27,783,151	-	732	83,295.6	8.24	44.03-unst	91.81	-0.045	Plasma membrane	
CreCNGC19	Cre5g_019,620.1	IV-A	4	5	22,012,270	22,033,591	-	777	89,294.76	9.43	46.42-unst	86.34	-0.215	Plasma membrane	
C. grandis CNGCs															
CgCNGC1.1	Cg9g028350.1	I	5	9	38,716,748	38,721,456	-	936	107,162.85	9.43	48.3-unst	91.4	-0.121	Plasma membrane	
CgCNGC1.2	Cg2g015410.1	I	1	2	19,778,512	19,781,575	+	299	34,216.96	9.22	47.85-unst	81.17	-0.285	Plasma membrane	
CgCNGC1.3	Cg9g028420.1	I	5	9	38,786,148	3,8,792,072	+	962	109,694.28	8.76	42.63-unst	87.76	-0.201	Plasma membrane	
CgCNGC1.4	Cg9g028570.1	I	5	9	39,116,699	39,120,252	+	677	78,034.42	8.65	39.24	94.91	-0.125	Plasma membrane	
CgCNGC1.5	Cg9g028370.1	I	6	9	38,743,875	38,747,793	-	594	68,616.61	6.89	43.85-unst	98.95	0.018	Plasma membrane	
CgCNGC1.6	Cg9g028360.1	I	11	9	38,727,773	38,740,199	-	1,289	147,329.05	8.7	39.32	98.98	0.066	Plasma membrane	
CgCNGC1.7	Cg5g040810.1	I	4	5	46,534,174	46,549,973	-	1,075	121,375.2	7.06	55.13-unst	85.26	-0.238	Plasma membrane	
CgCNGC2.1	Cg6g025480.1	IV-B	7	6	23,347,654	23,352,404	+	714	81,978.69	9.51	48.41-unst	93.1	-0.023	Plasma membrane	
CgCNGC2.2	Cg6g025470.1	IV-B	6	6	23,334,786	23,338,688	-	668	76,781.88	9.47	52.18-unst	100.24	0.039	Plasma membrane	
CgCNGC2.3	Cg9g020840.1	IV-B	4	9	30,004,781	30,012,835	+	729	83,109.01	6.06	38.86	101.09	-0.06	Plasma membrane	
CgCNGC2.4	Cg9g024210.1	IV-B	5	9	34,705,798	34,717,552	+	817	93,522.72	6.74	35.61	96.4	-0.122	Plasma membrane	
CgCNGC2.5	CgUng003220.1	IV-B	5	un	9,223,020	9,234,347	+	817	93,305.41	6.72	35.87	96.04	-0.13	Plasma membrane	
CgCNGC2.6	Cg1g026270.1	IV-B	5	1	28,354,084	28,359,345	+	822	94,163.21	6.33	39.91	96.19	-0.166	Plasma membrane	
CgCNGC4.1	Cg2g021840.1	IV-B	7	2	28,670,937	28,676,132	+	696	80,774.71	8.86	55.31-unst	89.21	-0.161	Plasma membrane	
CgCNGC4.2	Cg3g002670.1	IV-B	5	3	4,661,834	4,672,023	-	834	95,075.26	7.35	34	99.74	-0.08	Plasma membrane	
CgCNGC4.3	Cg4g003380.1	IV-B	5	4	3,087,474	3,110,776	-	1,020	115,444.86	6.33	47.72-unst	86.18	-0.232	Plasma membrane	
CgCNGC4.4	Cg2g044770.1	IV-B	6	2	51,339,028	51,343,678	+	780	89,204.05	6.22	45.62-unst	90.47	-0.231	Plasma membrane	
CgCNGC4.5	Cg4g002860.1	IV-B	5	4	2,629,924	2,633,950	+	885	99,323.22	7.62	44.8-unst	98.17	-0.122	Plasma membrane	
CgCNGC4.6	Cg4g007830.1	IV-B	5	4	8,156,335	8,162,186	+	884	99,796.39	7.57	37.6	95.37	-0.155	Plasma membrane	
CgCNGC5	Cg4g024210.1	II	5	4	28,735,763	28,743,584	+	734	84,009.72	9.04	49.85-unst	87.56	-0.171	Plasma membrane	
CgCNGC7	Cg1g021430.1	II	5	1	19,715,772	19,722,167	-	747	85,912.18	9.25	46.4-unst	93.82	-0.137	Plasma membrane	
CgCNGC8	Cg1g021420.1	II	5	1	19,706,850	19,712,083	-	749	86,209.43	9.22	50.26-unst	86.28	-0.189	Plasma membrane	
CgCNGC10	Cg9g028390.1	I	5	9	38,755,211	38,760,218	-	710	81,642.58	9.21	46.82-unst	88.98	-0.134	Plasma membrane	
CgCNGC15.1	Cg8g020040.1	III	7	8	17,731,791	17,735,688	+	733	83,844.22	9.61	45.47-unst	94.69	-0.153	Plasma membrane	
CgCNGC15.2	Cg8g020050.1	III	5	8	17,741,847	17,745,309	+	698	80,206.9	9.3	51.75-unst	92.18	-0.148	Plasma membrane	
CgCNGC15.3	Cg6g001850.2	III	6	6	3,649,106	3,652,936	+	705	81,198.2	9.21	52.81-unst	90.43	-0.103	Plasma membrane	
CgCNGC16	Cg5g004760.1	III	4	5	3,321,460	3,324,100	+	604	69,207.65	7.93	50.59-unst	93.81	-0.102	Plasma membrane	
CgCNGC17	Cg4g022550.1	III	5	4	26,987,542	26,992,837	-	725	83,522.38	9.35	41.65-unst	90.64	-0.213	Plasma membrane	
CgCNGC18	Cg2g006290.1	III	7	2	5,624,043	5,627,541	+	732	83,366.68	8.38	44.03-unst	91.53	-0.053	Plasma membrane	
CgCNGC19	Cg8g017160.1	IV-A	6	8	14,510,599	14,531,413	-	775	89,042.58	9.52	45.27-unst	86.69	-0.222	Plasma membrane	
A. buxfolia CNGCs															
AbuCNGC1.1	Abu9g_022,130.1	I	5	9	27,069,259	27,073,875	-	939	107,083.9	9.52	47.33-unst	93.51	-0.11	Plasma membrane	
AbuCNGC1.2	Abu2g_018,710.2	I	6	2	16,588,226	16,593,034	-	661	76,601.95	9.5	46.36-unst	94.09	-0.034	Plasma membrane	
AbuCNGC1.3	Abu2g_018,830.1	I	1	2	16,897,414	16,899,525	-	286	33,628.85	9.7	41.71-unst	84.81	-0.31	Plasma membrane	
AbuCNGC1.4	Abu8g_000,710.1	I	4	8	704,278	706,789	+	522	60,395.7	8.92	51.94-unst	92.61	-0.163	Plasma membrane	
AbuCNGC1.5	Abu9g_022,350.5	I	3	9	27,365,334	27,375,225	+	557	63,987.2	7.85	39.53	98.11	0.042	Plasma membrane	
AbuCNGC1.6	Abu9g_022,190.1	I	6	9	27,159,605	27,165,397	+	969	110,649.32	8.54	46.29-unst	88.73	-0.19	Plasma membrane	
AbuCNGC1.7	Abu9g_022,320.1	I	5	9	27,342,484	27,349,932	+	673	77,463.98	8.77	38.57	94.9	-0.103	Plasma membrane	
AbuCNGC1.8	Abu9g_022,150.1	I	12	9	27,113,191	27,121,549	-	1,181	136,159.61	8.58	39.59	94.56	0.037	Plasma membrane	
AbuCNGC1.9	Abu9g_022,140.1	I	11	9	27,095,048	27,108,466	-	1,335	152,436.23	8.58	39.62	100.53	0.092	Plasma membrane	
AbuCNGC2.1	Abu6g_000,500.2	IV-B	6	6	474,996	478,912	+	668	76,594.54	9.36	51.29-unst	98.79	0.036	Plasma membrane	
AbuCNGC2.2	Abu9g_015,870.2	IV-B	5	9	21,464,048	21,475,242	+	814	93,256.41	6.34	38.23	97.71	-0.113	Plasma membrane	
AbuCNGC2.3	Abu1g_022,970.1	IV-B	5	1	30,308,320	30,313,480	+	822	94,449.46	6.02	40.74-unst	95.94	-0.178	Plasma membrane	
AbuCNGC4.1	Abu4g_000,580.1	IV-B	7	4	929,289	934,439	-	693	80,470.5	9.02	55.04-unst	89.31	-0.162	Plasma membrane	
AbuCNGC4.2	Abu3g_004,740.1	IV-B	3	3	8,031,152	8,078,661	-	886	100,06.73	8.53	34.86	92.02	-0.243	Plasma membrane	
AbuCNGC4.3	Abu4g_010,210.1	IV-B	6	4	15,460,939	15,467,104	+	640	73,120.55	7.35	35.41	100.97	-0.021	Plasma membrane	
AbuCNGC4.4	Abu2g_002,010.1	IV-B	5	2	1,267,031	1,275,718	-	785	89,829.7	7.01	38.86	88.55	-0.256	Plasma membrane	
AbuCNGC4.5	Abu4g_010,650.1	IV-B	5	4	15,888,308	15,892,727	-	888	99,850.72	8.12	43.69-unst	96.84	-0.153	Plasma membrane	
AbuCNGC4.6	Abu4g_007,510.2	IV-B	5	4	13,188,706	13,194,715	-	884	99,691.1	6.79	38.84	94.81	-0.167	Plasma membrane	
AbuCNGC5	Abu4g_014,390.1	II	5	4	19,300,156	19,309,549	-	732	83,809.62	9.16	50.51-unst	87.93	-0.163	Plasma membrane	
AbuCNGC7	Abu1g_017,150.1	II	5	1	17,212,858	17,217,425	-	747	85,984.47	9.39	46.79-unst	94.49	-0.14	Plasma membrane	
AbuCNGC8	Abu1g_017,140.1	II	5	1	17,201,248	17,206,481	-	749	86,207.42	9.11	50.72-unst	86.14	-0.179	Plasma membrane	
AbuCNGC10	Abu9g_022,160.1	I	5	9	27,123,220	27,128,187	-	710	81,674.45	9.01	47.61-unst	89.8	-0.139	Plasma membrane	
AbuCNGC13	Abu5g_004,430.1	I	4	5	3,240,422	3,244,666	+	723	83,110.2	8.86	49.86-unst	88.74	-0.154	Plasma membrane	
AbuCNGC15.1	Abu8g_008,400.1	III	6	8	14,287,103	14,290,189	+	632	71,188.48	9.2	43.22-unst	98.68	0.013	Plasma membrane	
AbuCNGC15.2	Abu8g_008,410.1	III	5	8	14,297,412	14,301,227	+	698	80,160.93	9.33	51.8-unst	92.88	-0.148	Plasma membrane	
AbuCNGC15.3	Abu6g_018,420.1	III	6	6	15,292,506	15,295,954	-	711	81,741.94	9.29	53.96-unst	92.85	-0.087	Plasma membrane	
AbuCNGC16.1	Abuscaffold_270_000,010.1	III	scaffold_270		408	2,260	+	473	54,718.45	6.66	56.95-unst	83.52	-0.325	Plasma membrane	
AbuCNGC16.2	Abu5g_041,550.1	III	1	5	50,800,866	50,803,218	-	482	55,785.78	6.62	55.91-unst	84.18	-0.298	Plasma membrane	
AbuCNGC17	Abu4g_015,360.1	III	5	4	20,050,861	20,056,336	+	724	83,556.57	9.38	39.93	91.7	-0.209	Plasma membrane	
AbuCNGC18	Abu2g_024,930.1	III	7	2	25,802,387	25,806,145	-	732	83,306.53	8.24	45.67-unst	90.6	-0.063	Plasma membrane	
AbuCNGC19	Abu5g_025,960.1	IV-A	4	5	29,492,412	29,513,177	+	777	89,414.99	9.41	43.36-unst	87.98	-0.211	Plasma membrane	
P. trifoliata CNGCs															
PtCNGC1.1	Pt9g018530.1	I	5	9	25,015,230	25,020,827	-	973	110,771.04	9.37	48.94-unst	93.85	-0.11	Plasma membrane	
PtCNGC1.2	Pt9g018460.1	I	7	9	25,083,546	25,090,484	+	1,032	117,365.49	8.68	44.31-unst	91.9	-0.174	Plasma membrane	
PtCNGC1.3	Pt9g018430.1	I	9	9	25,179,672	25,187,070	+	1,250	143,552.89	8.45	42.96-unst	93.27	-0.072	Plasma membrane	
PtCNGC1.4	Pt9g018500.1	I	11	9	25,043,857	2,5,051,963	-	1,182	136,323.92	8.82	44.89-unst	93.67	0	Plasma membrane	
PtCNGC1.5	Pt9g018510.1	I	8	9	25,029,556	25,039,658	-	1,024	116,524.29	8.57	36.37	98.81	0.144	Plasma membrane	
PtCNGC2.1	Pt6g003400.1	IV-B	7	6	444,230	489,519	-	713	81,948.83	9.54	47.3-unst	94.73	0.003	Plasma membrane	
PtCNGC2.2	PtUn034160.1	IV-B	7	un	66,243,252	66,247,985	+	714	82,071.86	9.54	48.32-unst	92.96	-0.019	Plasma membrane	
PtCNGC2.3	Pt6g003390.1	IV-B	6	6	493,761	500,211	+	709	81,219.32	9.6	49.75-unst	102.01	0.067	Plasma membrane	
PtCNGC2.4	Pt6g003410.1	IV-B	5	6	476,681	480,770	+	664	76,206.35	9.53	50.54-unst	101.15	0.037	Plasma membrane	
PtCNGC2.5	Pt7g003350.3	IV-B	3	7	3,976,926	3,995,696	-	936	106,962.74	6.71	43.4-unst	84.49	-0.396	Plasma membrane, cytoplasmic, nuclear	
PtCNGC2.6	Pt9g012630.1	IV-B	5	9	14,547,685	14,559,074	+	816	93,222.34	6.86	35.94	95.68	-0.13	Plasma membrane	
PtCNGC4.1	Pt1g012590.1	IV-B	7	1	17,530,718	17,536,236	+	699	81,296.24	8.69	58.11-unst	89.24	-0.177	Plasma membrane	
PtCNGC4.2	PtUn009010.1	IV-B	5	un	15,359,017	15,369,473	-	832	94,926	7.59	34.09	99.39	-0.1	Plasma membrane	
PtCNGC4.3	Pt1g005510.1	IV-B	6	1	2,792,308	2,798,621	-	633	72,391.96	8.98	35.92	101.65	0.005	Plasma membrane	
PtCNGC4.4	Pt2g029140.1	IV-B	5	2	29,148,344	29,152,343	+	790	90,218.22	6.62	43.24-unst	89.1	-0.215	Plasma membrane	
PtCNGC4.5	Pt1g006070.1	IV-B	5	1	2,340,521	2,345,295	+	887	99,790.77	7.92	44.34-unst	98.6	-0.124	Plasma membrane	
PtCNGC4.6	Pt1g002920.1	IV-B	5	1	5,000,257	5,006,083	+	884	99,733.32	6.81	39.2	96.14	-0.141	Plasma membrane	
PtCNGC5	Pt1g019810.1	II	5	1	26,803,045	26,810,973	+	733	83,748.49	9.13	50.29-unst	88.36	-0.16	Plasma membrane	
PtCNGC7	Pt7g005810.1	II	4	7	8,049,190	8,059,979	+	1,061	121,091.84	9.18	44.54-unst	93.62	-0.156	Plasma membrane	
PtCNGC8	Pt7g005820.1	II	5	7	8,063,471	8,068,726	+	749	86,208.44	9.26	50-unst	86.27	-0.185	Plasma membrane	
PtCNGC10	Pt9g018490.1	I	5	9	25,053,675	25,058,703	-	710	81,507.37	9.09	46.31-unst	90.35	-0.115	Plasma membrane	
PtCNGC13	Pt3g033630.1	I	5	3	40,975,639	40,980,331	-	724	83,351.69	9.14	52.09-unst	89.97	-0.165	Plasma membrane	
PtCNGC14	Pt1g001460.1	III	1	1	615,563	619,051	+	575	66,098.6	8.35	46.52-unst	82.74	-0.333	Plasma membrane	
PtCNGC15.1	Pt8g007980.1	III	6	8	6,803,620	6,807,501	+	686	78,923.15	9.43	51.85-unst	89.21	-0.234	Plasma membrane	
PtCNGC15.2	Pt8g007990.1	III	6	8	6,812,971	6,817,286	+	696	80,231.95	9.31	50.7-unst	92.45	-0.157	Plasma membrane	
PtCNGC15.3	Pt6g018010.1	III	6	6	14,936,563	14,940,184	-	711	81,804.94	9.29	53.44-unst	89.82	-0.122	Plasma membrane	
PtCNGC16	Pt3g005720.1	III	4	3	3,690,317	3,693,148	+	604	69,139.53	7.93	50.71-unst	93.33	-0.109	Plasma membrane	
PtCNGC17	Pt1g020720.1	III	5	1	26,189,592	26,195,161	-	725	83,315.1	9.16	40.71-unst	90.77	-0.194	Plasma membrane	
PtCNGC18	Pt2g007780.1	III	7	2	5,133,162	5,136,635	+	732	83,451.7	8.24	43.42-unst	91	-0.068	Plasma membrane	
PtCNGC19	Pt3g017600.1	IV-A	4	3	19,663,677	19,685,454	-	777	89,598.1	9.43	45.22-unst	85.58	-0.237	Plasma membrane	

The protein length of CreCNGCs ranged from 371–1513aa, molecular weight (MW) ranged from 43.15–173.29 (KDa), Isoelectric point (PI) ranged from 6.33–9.44, 21 CreCNGCs proteins were unstable as they have II above 40. GRAVY values of 24 CreCNGCs were negative indicating that maximum proteins were hydrophilic. Only CreCNGC1.3 was localized in the plasma membrane and nuclear compartments while the rest were found to be localized in the plasma membrane.

CgCNGCs have protein lengths ranging from 299–1289aa, molecular weight (MW) ranging from 34.21–147.32 (KDa), Isoelectric point (PI) ranging from 6.06–9.52, Most of the proteins (22) of *C. grandis* were unstable as these proteins have II greater than 40. GRAVY values *for* 27 proteins of *C. grandis* were negative suggesting that these proteins were hydrophilic. All of the CgCNGCs were found to be localized in the plasma membrane.

*A. buxfolia* had protein length ranging from 286–1335aa, molecular weight (MW) ranging from 33.62–766.01 (KDa), Isoelectric point (PI) ranging from 6.02–9.7, Instability index (II) was above 40 for 21 proteins of *A. buxfolia* revealing that most of the proteins were unstable in the test tube. 24 proteins of *A. buxfolia* were hydrophilic as their GRAVY values were negative while 7 proteins were hydrophobic as their GRAVY values were positive. Results of subcellular localization demonstrated that all AbuCNGC proteins were found to be present in the plasma membrane.

*P. trifoliata*’s protein length ranged from 575–1250aa, molecular weight (MW) ranged from 12.10–143.55 (KDa) for *P. trifoliata*, Isoelectric point (PI) ranged from 6.62–9.54, Instability index (II) was above 40 for 25 proteins of *P. trifoliata* suggesting that most proteins were unstable. 25 PtCNGC proteins were hydrophilic because GRAVY values for these proteins were negative. For *P. trifoliata PtCNGC2.5* was localized in the plasma membrane as well as cytoplasmic and nuclear compartments while the rest were localized in the Plasma membrane. Hence, we can conclude that most of the proteins of *Citrus Spp.* were basic, unstable, hydrophilic, and localized in the Plasma membrane. The Citrus CNGC proteins that were stable can be used as a biomarker for further studies.

### 3.3 Phylogenetic analysis

**FIGURE 3:**
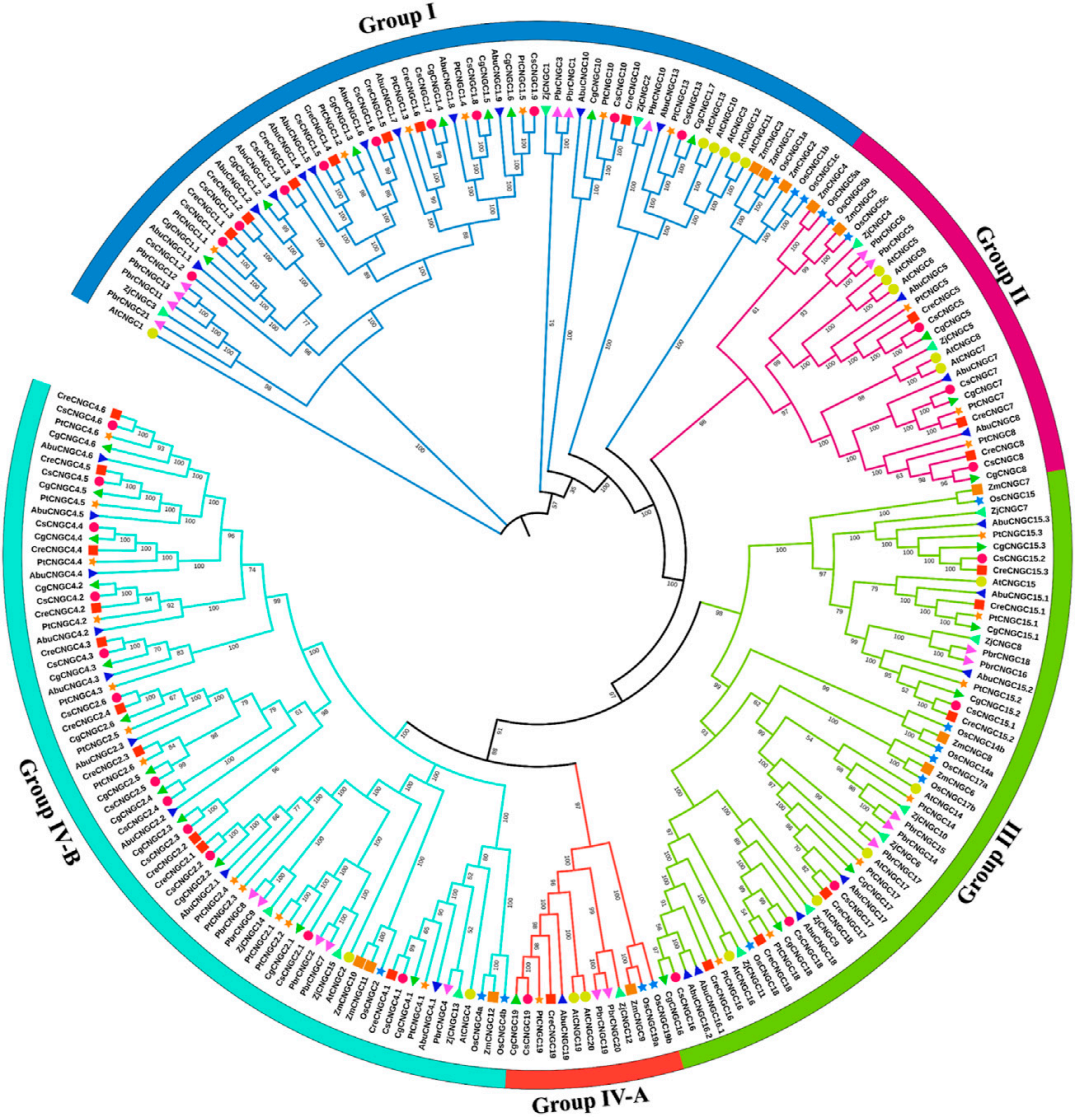
Phylogenetic relationship among AtCNGCs, OsCNGCs, ZjCNGCs, PbrCNGCs, ZmCNGCs, CsCNGCs, CreCNGCs, CgCNGCs, AbuCNGCs and PtCNGCs. The Multiple Sequence Alignment (MSA) has been done by using ClustalW. To build a phylogenetic tree, the IQ tree was utilized using the Maximum Likelihood method with 1,000 bootstrap replicates. Group names are indicated in front of each group. Different symbols are used to represent particular plants.

In total, 20 *AtCNGCs, 16 OsCNGCs, 12 ZmCNGCs, 21 PbrCNGCs, 15 ZjCNGCs, 32 CsCNGCs, 27 CreCNGCs, 30 CgCNGCs, 31 AbuCNGCs*, and 30 *PtCNGCs* genes were classified into four groups and the fourth group was further classified into two sub-groups, I, II, III, IV-A, IV-B each containing the different number of members. The maximum number of members were present in Group IV (84 members) divided into the clade of Group IV-B with 71 members: two members from *A. thaliana* (*AtCNGC2* and *4*), three from *O. sativa* (*OsCNGC2*, *4a* and *4b*), three from *Z. mays* (Z*mCNGC10*,*11* and *12*), five from *P. bretschneideri* (*PbrCNGC2*, *4*, *7*, *8* and *9*) three from *Z. jujuba* (*ZjCNGC13*, *14* and *15*), *12* from *C. sinensis* (*CsCNGC4.1-4.6* and *CsCNGC2.1-2.6*), 10 from *C. recticulata* (*CreCNGC4.1-4.6* and *CreCNGC2.1-2.4*), 12 from *C. grandis* (*CgCNGC4.1-4.6* and *CgCNGC2.1-2.6*), 9 from *A. buxfolia* (*AbuCNGC4.1-4.6*, *AbuCNGC2.1-2.3*) and 12 from *P. trifoliata* (*PtCNGC4.1-4.6* and *PtCNGC2.1-2.6*) and Group IV-A with 13 members: two from *A. thaliana* (*AtCNGC19* and *20*), two from *O. sativa* (*OsCNGC19a* and *19b*), one from *Z. mays* (*ZmCNGC9*), two from *P. bretschneideri* (*PbrCNGC19* and *20*), one from *Z. jujuba* (*ZjCNGC12*), one from *C. sinensis* (*CsCNGC19*), one from *C. recticulata* (*CreCNGC19*), one from *C. grandis* (*CgCNGC19*), one from *A. buxfolia* (*AbuCNGC19*) and one from *P. trifoliata* (*PtCNGC19*). The minimum number of members present in the clade of group II with 29 members two from *Z. mays* (*ZmCNGC4* and *ZmCNGC5*), three from *O. sativa* (*OsCNGC5a, OsCNGC5b,* and *OsCNGC5c*), two from *Z. jujube* (*ZjCNGC4* and *ZjCNGC5*), two from *P. bretschneideri* (*PbrCNGC5* and *PbrCNGC6*), five from *A. thaliana* (*AtCNGC5, AtCNGC6, AtCNGC7, AtCNGC8,* and *AtCNGC9*), three from *C. sinensis* (*CsCNGC5, CsCNGC7,* and *CsCNGC8*), three from *C. recticulata* (*CreCNGC5, CreCNGC7,* and *CreCNGC8*), three from *C. grandis* (*CgCNGC5, CgCNGC7,* and *CgCNGC8*), three from *A. buxfolia* (*AbuCNGC5*, *AbuCNGC7,* and *AbuCNGC8*), three from *P. trifoliata* (*PtCNGC5, PtCNGC7,* and *PtCNGC8*). The number of members in other groups was also different as Group I had 66 members and Group III had 56 members in total (Figure 3).

*CNGCs* from every group shared a clade with *Arabidopsis CNGC* members that are a dicot, which demonstrates that *CNGCs* emerged after the divergence of monocots and dicots. The close association of CNGC members in Citrus *Spp.* with AtCNGCs demonstrates that these are orthologs of CNGCs in *Arabidopsis*. Members of the same group might have similar structures and functions. The results of phylogenetic analysis of CNGCs in Citrus *Spp.* were different than those in *A. thaliana, O. sativa, T. aestivum, N. tobaccum, B. oleracea, B. rapa, P. bretschneideri, Z. jujuba* as current analysis revealed that Group IV clade was largest with 84 members in total and the clade of group II was smallest with 29 members. The number of members in group IV was almost consistent with the previously reported number of members in *Z. mays,* which had 86 members in group IV. The minimum number of members present in the clade of group I was 25. Overall, the number of members was different in each group as compared to previously reported CNGC members in other plants.

### 3.4 Gene structure and conserved motif analysis

Gene structure analysis revealed that members from each subspecies are having their own set of exons and introns. Exons that belong to group I of *CsCNGC* ranged from 6 to 17 while exons that belong to group I of *AtCNGCs* ranged from 7 to 9. Exons that belong to group II of *CsCNGC* were 7 while exons that belong to group II of *AtCNGCs* ranged from 6 to 9. Exons that belong to group III of *CsCNGC* and *AtCNGC* ranged from 6 to 7. Exons that belong to group IV-A of *CsCNGC* were 12 while exons that belong to group IV-A of *AtCNGC* ranged from 10 to 11. Exons that belong to group IV-B of *CsCNGC* ranged from 7 to 14 while exons that belong to group IV-B of *AtCNGC* ranged from 8 to 9.

**FIGURE 4:**
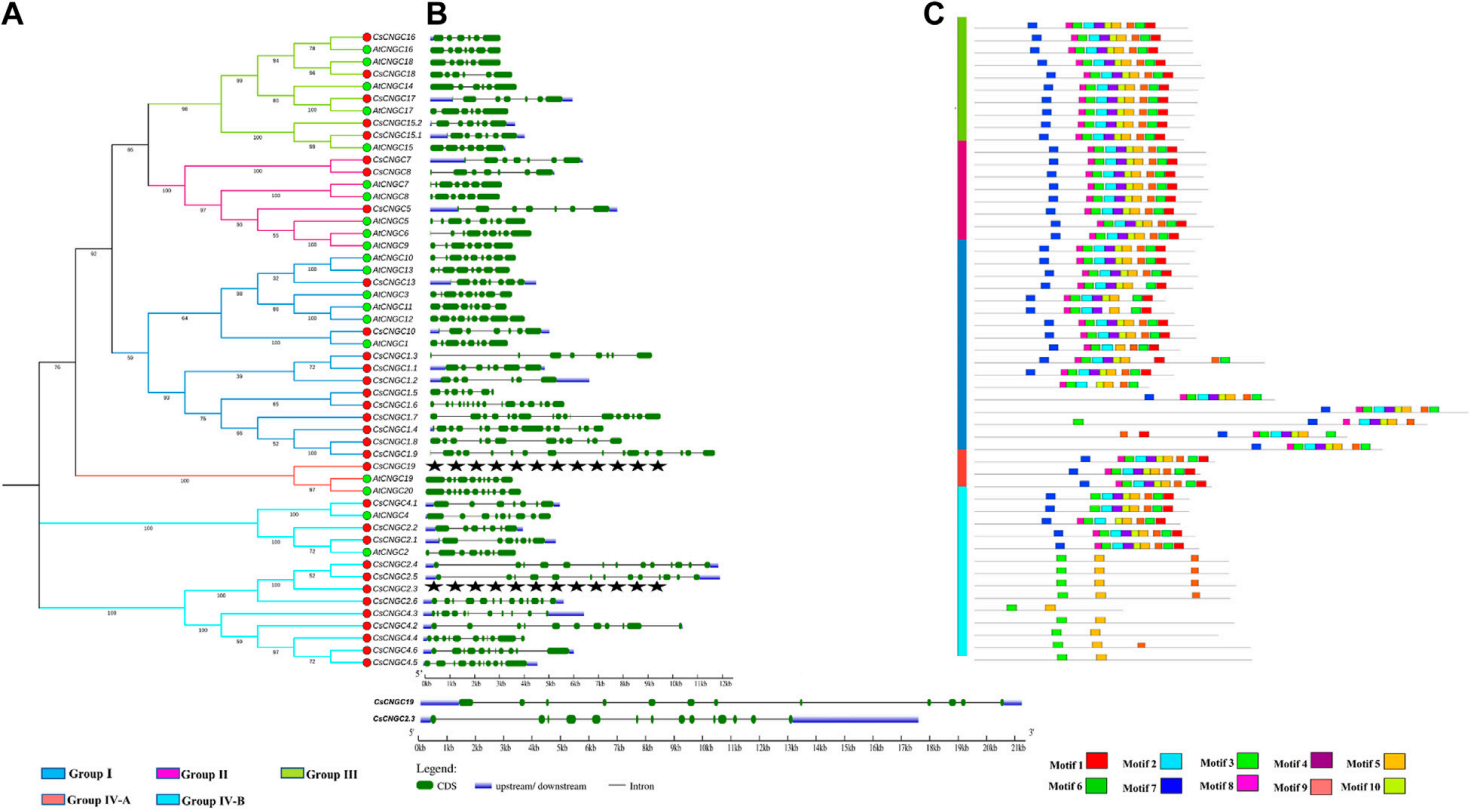
**(A)** Phylogenetic tree constructed at MEGA 7.0 based on Neighbor-Joining method with a bootstrap value of 1,000 replicates using protein sequences of AtCNGCs and CsCNGCs. Red colored circles represent CsCNGCs and green colored circles represent AtCNGCs. **(B)** Gene structures of *AtCNGC* and *CsCNGC were* determined by using GSDS v2.0. **(C)** Representation of conserved motifs in AtCNGCs and CsCNGCs determined by using MEME suite.

Ten motifs were identified in CsCNGCs and named motif 1 to motif 10. Motif 1 represents a combination of the Calmodulin binding motif (CaMB) and motif for the IQ domain. Motif 6 represents the hinge motif, while motif 9 represents the PBC motif. Both these motifs together constitute the cNMP/Cyclic nucleotide-binding domain (CNBD). Other motifs are responsible for unknown functions. The gene structure and logo of conserved motifs of *C. Sinensis* are given in (Figure 4, Supplementary Figure S5).

Group I of *CreCNGCs* contained exons ranging from 4 to 17, 5 to 7 exons exist in group II of *CreCNGC*, exons that exist in group III of *CreCNGCs* were 6–8, and 12 exons were present in group IV-A of *CreCNGCs* and 7 to 13 exons were present in group IV-B of *CreCNGCs*. Motif 5 represents the PBC motif and motif 2 contains the hinge motif, CaMB motif, and motif for the IQ domain. Other motifs were representing motifs of unknown function (Supplementary Figures S6A,B).

Group I of *CgCNGC* contained 4 to 17 exons, group II of *CgCNGC* contained 7 exons, group III of *CgCNGC* contained 6 to 8, and group IV-A of *CgCNGC* contained 12 exons and group IV-B of *CgCNGC* contained 7 to 14 exons. Motif 3 represents a combination of CaMB motif and motif for IQ-domain, motif 5 represents hinge region motif and motif 7 represents PBC motif (Supplementary Figures S7A,B).

In *AbuCNGC* exons of group I were ranging from 4 to 18, exons of group II were ranging from 6 to 7, exons of group III were ranging from 4 to 7, exons of group IV-A 12, and exons of group IV-B were ranging from 7 to 14. Motif 3 represents a combination of CaMB motif and motif for IQ-domain, motif 8 represents PBC motif and motif 2 contains hinge motif (Supplementary Figures S8A,B).

Exon number for group I of *PtCNGC* ranged from 6 to 16, exon number for group II of *PtCNGC* ranged from 7 to 12, exon number for group III of Pt*CNGC* ranged from 6 to 9, and exon number for group IV-A of *PtCNGC* were 12 and exon number for group IV-B of Pt*CNGC* ranged from 7 to 18. Motif 2 represents the CaMB motif and motif for the IQ domain, motif 3 represents the Cyclic nucleotide-binding domain that contains both PBC and hinge motif. The representation of motifs and logo of conserved motifs of *P. trfoliata* is displayed (Supplementary Figures S9A,B).

Hence, the PBC motif, hinge motif, CaMB motif, and motif for the IQ domain was conserved in 5 Citrus *Spp.* indicating that genes identified in the current study are truly *CNGC genes*.

### 3.5 Chromosomal mapping

**FIGURE 5:**
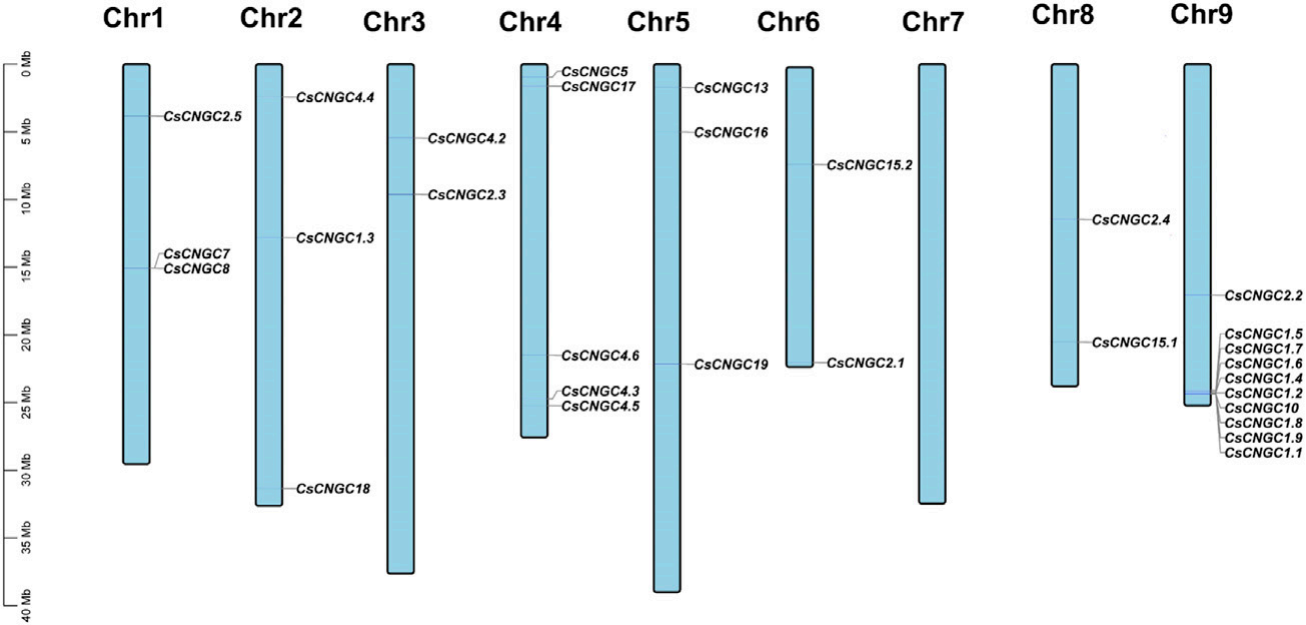
Distribution of *CsCNGC* genes on chromosomes. Genes get mapped on chromosomes based on information available at the Citrus pan-genome to breeding database. Chromosome numbers are indicated at the top of each chromosome. The scale is given in Megabases (Mb).

In *C. sinensis* 32 genes were distributed unevenly on 8 out of 9 chromosomes*. C. sinensis* had maximum genes (10) at chromosome 9, minimum genes (2) at chromosomes 3, 6, and 8, and there was no gene on chromosome 7. The distribution of *CsCNGC* on chromosomes is given in (Figure 5).

27 genes were mapped unevenly at 8 out of 9 chromosomes in *C. recticulata.* In *C. recticulata* maximum genes (8) were present at chromosome 9, minimum genes (1) were present at chromosome 3, and no gene was present at chromosome 7 (Supplementary Figure S10). In *C. grandis* chromosome 9 carried maximum genes (8), chromosome 3 carried minimum genes (1), and none of the genes was present on chromosome 7 (Supplementary Figure S11). In *A. buxfolia* chromosome 9 contained maximum genes (8), chromosome 3 contained minimum genes (1) and none of the genes was present on chromosome 7 (Supplementary Figure S12). In *P. trifoliata* there were maximum genes (7) present at chromosome 9 and chromosome 1, there were minimum genes (2) present at chromosomes 2 and 8 and there was no gene present at chromosomes 4 and 5 (Supplementary Figure S13). Thus, it can be inferred that *CNGC* genes were distributed unevenly at 8 out of 9 chromosomes in Citrus *Spp.* except for *P. trifoliata* in which genes were distributed at 7 out of 9 chromosomes.

### 3.6 Gene duplication events

The duplication pairs resulting from segmental duplication in *C. sinensis* include *CsCNGC2.3/CsCNGC2.4*, *CsCNGC2.3/CsCNGC2.5, CsCNGC2.3/CsCNGC2.6, CsCNGC2.4/CsCNGC2.5, CsCNGC2.4/CsCNGC2.6, CsCNGC2.5/CSCNGC2.6*. The gene pairs that were tandemly duplicated in *C. sinensis* include *CsCNGC1.1/CsCNGC1.8, CsCNGC1.8/CsCNGC1.9, CsCNGC2.1/CsCNGC2.2, CsCNGC7/CsCNGC8*. The gene pairs of *A. thaliana* CNGCs that were tandemly duplicated include *AtCNGC3/AtCNGC11, AtCNGC7/AtCNGC8, AtCNGC11/AtCNGC12, AtCNGC19/AtCNGC20*. The gene pairs of *A. thaliana* CNGCs that were segmentally duplicated include *AtCNGC3/AtCNGC13, AtCNGC5/AtCNGC8, AtCNGC6/AtCNGC7, AtCNGC6/AtCNGC9, AtCNGC10/AtCNGC13, AtCNGC14/AtCNGC17*. Genes were duplicated segmentally as well as tandemly in both *C. sinensis* and *A. thaliana* indicating that both segmental and tandem duplications are involved in the expansion of *CsCNGC* genes. Moreover, the rate of non-synonymous substitutions (Ka), rate of synonymous substitutions (Ks), Ka/Ks, and duplication time (MYA) were calculated. The Ks of 6 segmental duplicates in *C. sinensis* ranged from 0.0118 to 0.8374, also Ks of 4 tandem duplicates ranged from 0.2099 to 2.0937, and duplication time of both segmental and tandem duplicates ranged from 0.89 MYA to 159 MYA. The Ka/Ks value of *CsCNGC1.1/CsCNGC1.8, CsCNGC2.1/CsCNGC2.2, CsCNGC2.3/CsCNGC2.4*, *CsCNGC2.3/CsCNGC2.6, CsCNGC2.4/CsCNGC2.5, CsCNGC2.4/CsCNGC2.6, CsCNGC2.5/CSCNGC2.6* was less than 1 indicating the occurrence of purifying selection in duplication of these genes. The Ka/Ks value of *CsCNGC1.8/CsCNGC1.9, CsCNGC2.3/CsCNGC2.5, CsCNGC7/CsCNGC8* was greater than 1 indicating the role of positive selection in duplication of these genes. Similarly, the Ks of 6 segmental duplicates in *A. thaliana* ranged from 0.2735 to 1.1835, and also the Ks value of 4 tandem duplicates ranged from 0.0504 to 0.9547, and the duplication time of both segmental and tandem duplicates ranged from 3.84 MYA to 90.20 million years ago (MYA) (Table 3).

**TABLE 3:** Ka, Ks, Ka/Ks values calculated for homologous gene pairs of *A. thaliana* and *C. sinensis*.

Gene 1	Gene 2	Ka	Ks	Ka/Ks	Duplication time (MYA)	Duplication Type
*AtCNGC3*	*AtCNGC11*	0.4516	0.9547	0.473,028,176	72.76,676,829	Tandem
*AtCNGC3*	*AtCNGC13*	0.4489	1.0099	0.444,499,455	76.97,408,537	Segmental
*AtCNGC5*	*AtCNGC8 *	0.4158	0.824	0.50,461,165	62.80,487,805	Segmental
*AtCNGC6*	*AtCNGC7*	0.3829	0.6195	0.618,079,096	47.2,179,878	Segmental
*AtCNGC6*	*AtCNGC9*	0.1566	0.3842	0.407,600,208	29.28,353,659	Segmental
*AtCNGC7*	*AtCNGC8*	0.1359	0.3399	0.399,823,477	25.9,070,122	Tandem
*AtCNGC10*	*AtCNGC13*	0.192	0.2735	0.702,010,969	20.84,603,659	Segmental
*AtCNGC11*	*AtCNGC12*	0.0167	0.0504	0.331,349,206	3.841,463,415	Tandem
*AtCNGC14*	*AtCNGC17*	0.6632	1.1835	0.560,371,779	90.20,579,268	Segmental
*AtCNGC19*	*AtCNGC20*	0.2291	0.2634	0.869,779,803	20.07,621,951	Tandem
*CsCNGC1.1*	*CsCNGC1.8*	1.59	2.0937	0.759,421,121	159.5,807,927	Tandem
*CsCNGC1.8*	*CsCNGC1.9*	1.8165	1.7537	1.035,810,002	133.6,661,585	Tandem
*CsCNGC2.1*	*CsCNGC2.2*	0.6555	0.8614	0.760,970,513	65.6,554,878	Tandem
*CsCNGC2.3*	*CsCNGC2.4*	0.0135	0.0238	0.567,226,891	1.81,402,439	Segmental
*CsCNGC2.3*	*CsCNGC2.5*	0.0343	0.0118	2.906,779,661	0.899,390,244	Segmental
*CsCNGC2.3*	*CsCNGC2.6*	0.6756	0.8021	0.842,288,991	61.13,567,073	Segmental
*CsCNGC2.4*	*CsCNGC2.5*	0.0343	0.0359	0.955,431,755	2.736,280,488	Segmental
*CsCNGC2.4*	*CsCNGC2.6*	0.6828	0.7726	0.883,769,091	58.88,719,512	Segmental
*CsCNGC2.5*	*CsCNGC2.6*	0.6592	0.8374	0.787,198,471	63.82,621,951	Segmental
*CsCNGC7*	*CsCNGC8*	0.2583	0.2099	1.230,585,993	15.99,847,561	Tandem

In *C. recticulata* gene pairs that were the product of segmental duplication include *CreCNGC2.2/CreCNGC2.4, CreCNGC2.3/CreCNGC2.4.* The gene pairs that were the product of tandem duplication include *CreCNGC2.2/CreCNGC2.3, CreCNGC7/CreCNGC8, and CreCNGC15.1/CreCNGC15.2.* Altogether 5 gene pairs were duplicated and among these 3 gene pairs were tandemly duplicated indicating the role of tandem duplication in the expansion of *CreCNGC* genes. The Ks of 2 segmental duplicates in *C. recticulata* were 0.50, also Ks of 3 tandem duplicates ranged from 0.05 to 0.56, and the duplication time of both segmental and tandem duplicates ranged from 4.23 MYA to 42.74 MYA. The Ka/Ks value of *CreCNGCs* was less than 1 indicating that purifying selection has occurred in this duplication event (Supplementary Table S3).

The segmentally duplicated gene pairs of *C. grandis* include *CgCNGC2.3/CgCNGC2.5, CgCNGC2.3/CgCNGC2.6, CgCNGC2.4/CgCNGC2.5, CgCNGC2.4/CgCNGC2.6, CgCNGC2.5/CgCNGC2.6.* The tandemly duplicated gene pairs include *CgCNGC1.5/CgCNGC1.6, CgCNGC2.1/CgCNGC2.2, CgCNGC2.3/CgCNGC2.4, CgCNGC7/CgCNGC8, CgCNGC15.1/CgCNGC15.2.* Overall, 10 gene pairs were duplicated and out of these 5 gene pairs were segmentally duplicated and 5 were tandemly duplicated indicating the equal contribution of both events in the expansion of *CgCNGC* genes. The Ks of 5 segmental duplicates in *C. grandis* ranged from 0.03 to 0.74, also Ks of 5 tandem duplicates ranged from 0.02 to 0.44, and the duplication time of both segmental and tandem duplicates ranged from 1.71 MYA to 69.20 MYA. The Ka/Ks value of five gene pairs was less than 1 indicating the role of purifying selection in the duplication of these genes. The Ka/Ks value of four gene pairs was less than 1 indicating the role of purifying selection in the duplication of these gene pairs and one gene pair (*CgCNGC2.3/CgCNGC2.4*) greater than 1 indicating the role of positive selection in the duplication of this gene pair (Supplementary Table S3).

The gene pairs that were segmentally duplicated in *A. buxfolia* include *AbuCNGC1.2/CreCNGC1.7, AbuCNGC1.2/AbuCNGC1.9, AbuCNGC1.3/AbuCNGC1.7, AbuCNGC1.3/AbuCNGC1.9, AbuCNGC2.2/AbuCNGC2.3.* Tandemly duplicated gene pairs include *AbuCNGC1.1/AbuCNGC1.8, AbuCNGC1.1/AbuCNGC10, AbuCNGC1.8/AbuCNGC1.9, AbuCNGC7/AbuCNGC8.* In total 9 gene pairs were duplicated and among these 5 gene pairs were segmentally duplicated and 4 were tandemly duplicated indicating the role of segmental duplication in the expansion of *AbuCNGC* genes. The Ks of 5 segmental duplicates in *A. buxfolia* ranged from 0.85 to 2.55, also Ks of 4 tandem duplicates ranged from 0.14 to 1.85, and the duplication time of both segmental and tandem duplicates ranged from 11.36 MYA to 195 MYA. The Ka/Ks value of eight gene pairs was less than 1 indicating the role of purifying selection in the duplication of these gene pairs. While the Ka/Ks value of only one gene pair (*AbuCNGC7/AbuCNGC8*) was greater than 1 indicating the role of positive selection in the duplication of this gene pair (Supplementary Table S3).

The duplicated gene pairs that arise from segmental duplication in *P. trifoliata* include *PtCNGC2.2/PtCNGC2.3, PtCNGC2.2/PtCNGC2.4, PtCNGC2.5/PtCNGC2.6, PtCNGC5/PtCNGC8.* The gene pairs that arise from tandem duplication include *PtCNGC1.4/PtCNGC1.5, PtCNGC2.1/PtCNGC2.3, PtCNGC2.1/PtCNGC2.4, PtCNGC2.3/PtCNGC2.4, PtCNGC14/PtCNGC1, PtCNGC15.1/PtCNGC15.2.* A total of 10 gene pairs were duplicated and among them, 4 gene pairs were segmentally duplicated and 6 were tandemly duplicated indicating the role of tandem duplication in the expansion of *PtCNGC* genes. Moreover, the rate of non-synonymous substitutions (Ka), rate of synonymous substitutions (Ks), Ka/Ks, and duplication time (MYA) were calculated. The Ks of 4 segmental duplicates in *P. trifoliata* ranged from 1.09 to 1.83, also Ks of 6 tandem duplicates ranged from 0.06 to 1.45, and the duplication time of both segmental and tandem duplicates ranged from 4.81 MYA to 111.25 MYA. Mostly gene pairs have Ka/Ks value of less than 1 indicating the role of purifying selection in the duplication of these gene pairs. While the Ka/Ks value of *PtCNGC1.4/PtCNGC1.5* was greater than 1 indicating the role of positive selection in the duplication of this gene pair (Supplementary Table S3).

### 3.7 Cis-regulatory elements/promoter analysis of citrus *Spp.*

To clearly understand the role of cis-regulatory elements (CREs) in *CsCNGCs, CreCNGCs, CgCNGCs, AbuCNGCs, and PtCNGCs,* and the cis-elements in 2 kb upstream of TSS were identified. The results suggested that cis-elements of four types were identified namely, hormone-responsive, light-responsive, stress-related cis-elements, and plant development-related cis-elements in *CsCNGCs, CreCNGCs, CgCNGCs, AbuCNGCs, PtCNGCs.*

**FIGURE 6:**
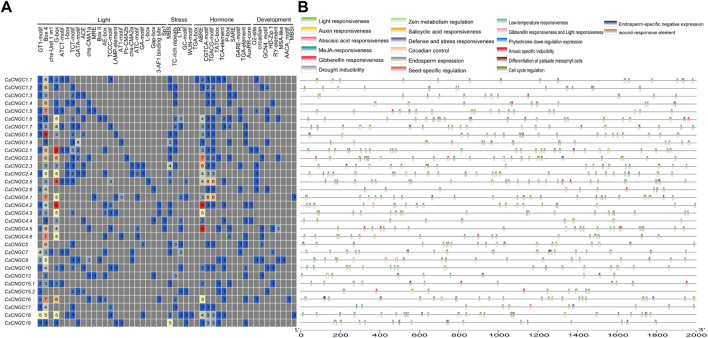
Cis-elements analysis done on promoter regions of *CsCNGC*. **(A)** The different colors and numbers represent the number of promoter elements in *CsCNGC* genes. **(B)** Colored bars represent cis-elements of different types and their locations in each *CsCNGC* gene. The types, numbers, and locations of cis-elements in promoter regions 2 kb upstream of *CsCNGC* genes were checked by using the PlantCare database.

It was observed that cis-elements responsible for light responsiveness were present abundantly in *CsCNGCs.* Overall, 24 cis-elements responsible for light responsiveness were determined out of which Box 4 element was present in 31 *CsCNGCs,* GT1-motif and G box elements were present in 22 *CsCNGCs* and 23 *CsCNGCs* and others were present in very few *CNGCs.* Among 11 hormone-related cis-elements, the ABRE element was present in 22 *CsCNGCs,* the CGTCA motif and TGACG motifs were present in 20 *CsCNGCs,* and 21 *CsCNGCs,* TCA element, and TATC box were present in 9 *CsCNGCs* and *11 CsCNGCs* and other hormone-related elements were present in very few *CsCNGCs.* Among 5 stress-related cis-elements, MBS element (drought inducible) was present in 16 *CsCNGCs,* TC-rich repeats element (defense responsive) was present in 13 *CsCNGCs* and LTR, GC motif, WUN motif was present in very few *CsCNGCs.* Out of 8 development-related cis-elements, GCN4_motif and circadian were present in 7 *CsCNGCs and* O2 site element was present in *9 CsCNGCs* and others were present in very few *CsCNGCs.* The results demonstrate that *CsCNGCs* are involved in plant growth, development, and response to abiotic stress. The graphical representation of the location and types of cis-elements present in *CsCNGCs* is given in (Figure 6).

Cis-elements responsible for light responsiveness were present abundantly in *CreCNGCs*. Overall, 27 cis-elements responsible for light responsiveness were determined out of which Box4 was present in 25 *CreCNGCs,* G box was present in 22 *CreCNGCs, and* GT1 motif was present in 17 *CreCNGCs* and others were present in very few *CreCNGCs*. Among 10 hormone-related cis-elements, ABRE was present in 18 *CreCNGCs,* TGACG motif was present in 17 *CreCNGCs,* TCA element was present in 14 *CreCNGCs* and others were present in very few *CreCNGCs.* Among 4 stress-related cis-elements, LTR was present in 12 *CreCNGCs*, MBS was present in 10 *CreCNGCs*, TC-rich repeats element was present in 9 *CreCNGCs, and* GCmotif was present in 4 *CreCNGCs.* Out of 6 development-related cis-elements RY element, O2 site, and GCN4 motif were present in 6 *CreCNGCs* and others were present in very few *CreCNGCs* (Supplementary Figure S14).

*CgCNGCs* also contained a number of light-responsive CREs. Overall, 24 cis-elements responsible for light responsiveness were determined out of which Box4 was present in 28 *CgCNGCs,* G box was present in 25 *CgCNGCs*, and GT1 motif was present in 22 *CgCNGCs* and others were present in very few *CgCNGCs.* Among 9 hormone-related cis elements ABRE was present in 23 *CgCNGCs,* CGTCA motif was present in 17 *CgCNGCs,* TGACG motif was present in 18 *CgCNGCs* and others were present in very few *CgCNGCs.* Among 5 stress-related cis-elements, MBS was present in 18 *CgCNGCs*, TC rich repeats element was present in 13 *CgCNGCs,* LTR was present in 11 *CgCNGCs* and others were present in very few *CgCNGCs.* Out of 7 development-related cis-elements, circadian was present in 5 *CgCNGCs,* GCN4 motif was present in 5 *CgCNGCs* and others were present in very few *CgCNGCs* (Supplementary Figure S15).

Cis-elements responsible for light responsiveness were present abundantly in *AbuCNGCs.* Overall, 24 cis-elements responsible for light responsiveness were determined out of which G box was present in 26 *AbuCNGCs,* Box 4 was present in 26 *AbuCNGCs, and* GT1 motif was present in 21 *AbuCNGCs,* TCT motif was present in 21 *AbuCNGCs* and others were present in very few *AbuCNGCs.* Among 9 hormone-related cis elements ABRE was present in 25 *AbuCNGCs,* TGACG motif and CGTCA motif were present in 16 *AbuCNGCs* and others were present in very few *AbuCNGCs.* Among 4 stress-related cis-elements, MBS was present in 17 *AbuCNGCs,* TC-rich repeats element was present in 10 *AbuCNGCs,* and others were present in very few *AbuCNGCs.* Among 6 development-related cis-elements, circadian was present in 7 *AbuCNGCs,* O2 site was present in *5 AbuCNGCs* and others were present in very few *AbuCNGCs* (Supplementary Figure S16).

Cis-elements responsible for light responsiveness were present abundantly in *PtCNGCs.* Overall, 24 cis-elements responsible for light responsiveness were determined out of which Box 4 was present in 25 *PtCNGCs,* G box was present in 21 *PtCNGCs,* and GT1 motif was present in 18 *PtCNGCs* and others were present in very few *PtCNGCs*. Among 10 hormone-related cis-elements, ABRE was present in 22 *PtCNGCs,* CGTCA motif and TGACG motif were present in 17 *PtCNGCs* and others were present in very few *PtCNGCs.* Among 5 stress-related cis-elements, MBS was present in 17 *PtCNGCs,* TC-rich repeats element was present in 11 *PtCNGCs,* LTR was present in 7 *PtCNGCs* and others are present in very few *PtCNGCs*. Among 7 development-related cis-elements, the RY element was present in 5 *PtCNGCs,* Circadian was present in 4 *PtCNGCs* and others were present in very few *PtCNGCs* (Supplementary Figure S17).

### 3.8 Pan-genome wide investigation of miRNAs targeting *CsCNGC* genes, protein-protein interaction, and gene ontology enrichment analysis

**FIGURE 7:**
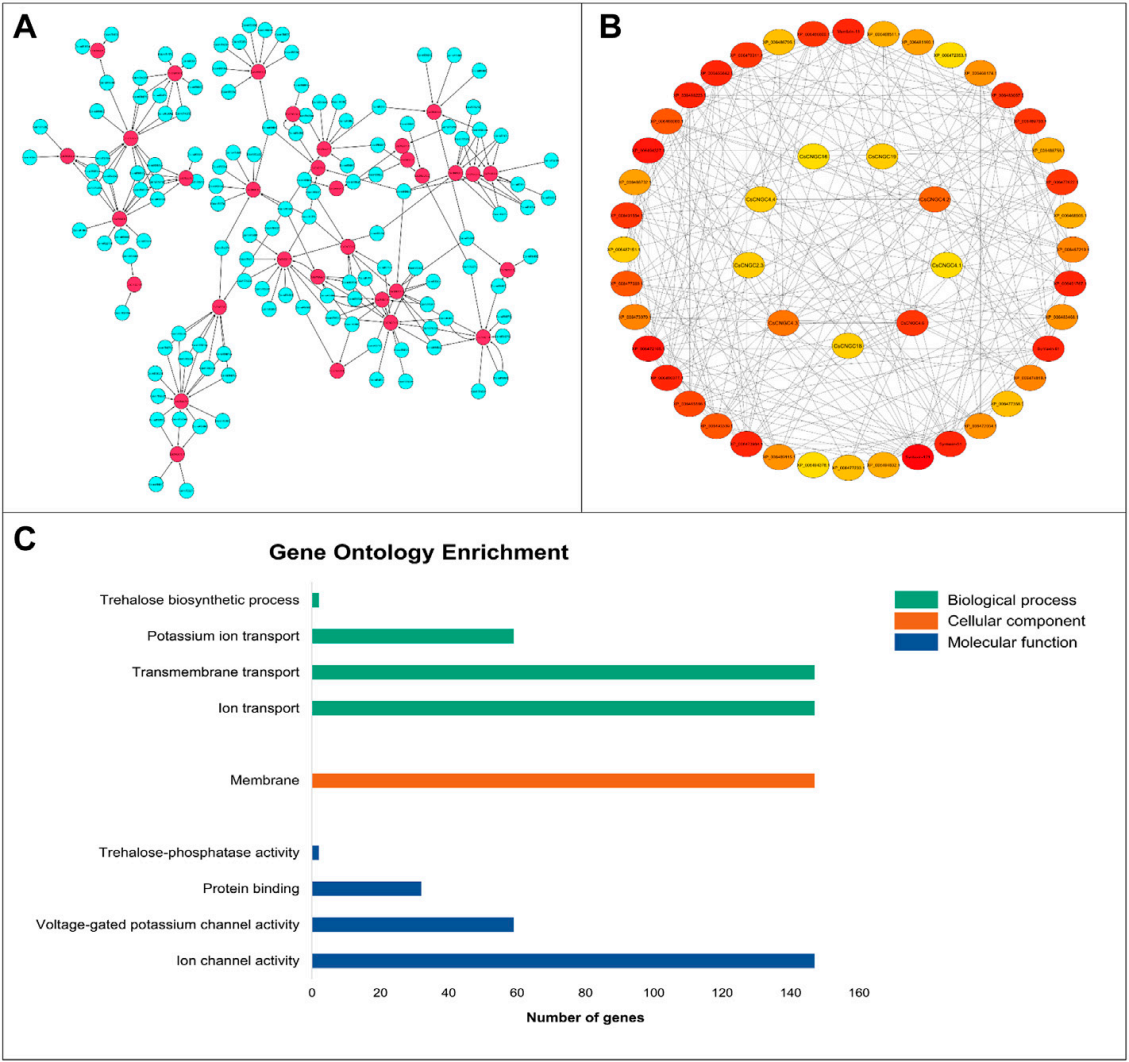
**(A)** Network representation of regulatory association among miRNAs and *CsCNGCs*. The network has been constructed by using Cytoscape. The miRNAs involved in regulating *CsCNGCs* are colored blue. *CsCNGC* genes are colored red and black colored lines represent the regulatory relationship. **(B)** Network showing the interactions among *CsCNGCs* and other protein members predicted using STRING database. The nodes are colored according to the degree of interactions. The red color is showing the protein has a higher level of connectivity with other members, orange-colored nodes have a relatively lesser level of interactions with other proteins while yellow-colored nodes have the least interactions with other proteins. **(C)** Gene ontology enrichment statistics graph, the green color bar represents biological processes, the orange color bar represents a cellular component, and the blue color bar represents the molecular function.

A total of 226 miRNAs were identified that targeted 32 *CsCNGCs* with expectation values ranging from 3.5 to 5 (Figure 7A). Only 1 miRNA was targeting *CsCNGC17* with an expectation value of 3.5, while 16 miRNAs were targeting *CsCNGC7* where all miRNAs have expectation value 5 except Csi-miRN925 with expectation value 4.5, 10 miRNAs were targeting *CsCNGC1.1*, *CsCNGC2.3* and *CsCNGC2.4*, 4 miRNAs were targeting *CsCNGC1.2*, 9 miRNAs were targeting *CsCNGC1.3, CsCNGC10* and *CsCNGC13*, 11 miRNAs were targeting *CsCNGC1.4* and *CsCNGC1.8,* 5 miRNAs were targeting *CsCNGC1.5*, *CsCNGC15.1* and *CsCNGC2.1*, 7 miRNAs were targeting *CsCNGC1.6* and *CsCNGC18*, 13 miRNAs were targeting *CsCNGC1.7, CsCNGC16* and *CsCNGC8*, 3 miRNAs were targeting *CsCNGC1.9, CsCNGC15.2, CsCNGC2.6, CsCNGC4.3* and *CsCNGC5*, 2 miRNAs were targeting *CsCNGC4.1, CsCNGC4.2* and *CsCNGC19,* 6 miRNAs were targeting *CsCNGC2.2,* 8 miRNAs were targeting *CsCNGC2.5, CsCNGC4.4* and *CsCNGC4.5*, 6 miRNAs were targeting *CsCNGC4.6*. Detailed information related to these miRNAs regulated *CsCNGCs* is given in (Supplementary Table S4). Among these miRNAs, most of them were responsible for inhibiting the cleavage of target transcript while only a few were involved in inhibiting the translation of target genes.

The PPI network of CsCNGC proteins was constructed to reveal the interaction among these proteins and related proteins (Figure 7B) to understand their degree of connectivity and ultimately their functional relativity. It has been shown that the highest degree of connectivity was shown by syntaxin-121, a protein from the *C. sinensis* plant, which suggests that this protein may have some functional connectivity with the CNGC proteins. Similarly, other proteins including Membrin-11 and some vesicle-associated membrane proteins (acc: XP_006479311.1) also showed a higher degree of interaction. Among CNGC members, CsCNGC4.6, CsCNGC4.2, and CsCNGC4.3 had higher interactions with other CNGC members as well as other related proteins. CsCNGC2.3, CsCNGC4.1, CsCNGC4.4, CsCNGC16, CsCNGC18 and CsCNGC19 had relatively lesser interactions. This level of connectivity reveals that these proteins might be involved in similar pathways thus regulating particular reactions and performing similar functions.

GO enrichment analyses were carried out on 5 Citrus *Spp.* to increase our understanding of the dynamic roles of CNGCs genes at the molecular level (Figure 7C; Supplementary Table S5). Based on GO analysis genes are classified into three major categories: biological process (BP), cellular component (CC), and molecular function (MF). Genes were mostly related to biological processes (4), molecular functions (4), and then cellular components (1). In the biological process, category 147 out of 150 genes were involved in ion transport (GO:0,006,811) and transmembrane transport (GO:0,055,085), 59 genes were involved in potassium ion transport (GO:0,006,813), and only 2 genes were involved in trehalose biosynthetic process (GO:0,005,992). In the cellular component category, 147 genes were mainly found in the membrane (GO:0,016,020) which is consistent with the subcellular localization prediction result. In the molecular function category, 147 genes were involved in ion channel activity (GO:0,005,216), 59 genes out of 150 are involved in voltage-gated potassium channel activity (GO:0,005,249), 32 genes in protein binding (GO:0,005,515), and only 2 genes were involved in the trehalose-phosphatase activity (GO:0,004,805).

### 3.9 Expression profiling of *C. sinensis* under drought stress

**FIGURE 8:**
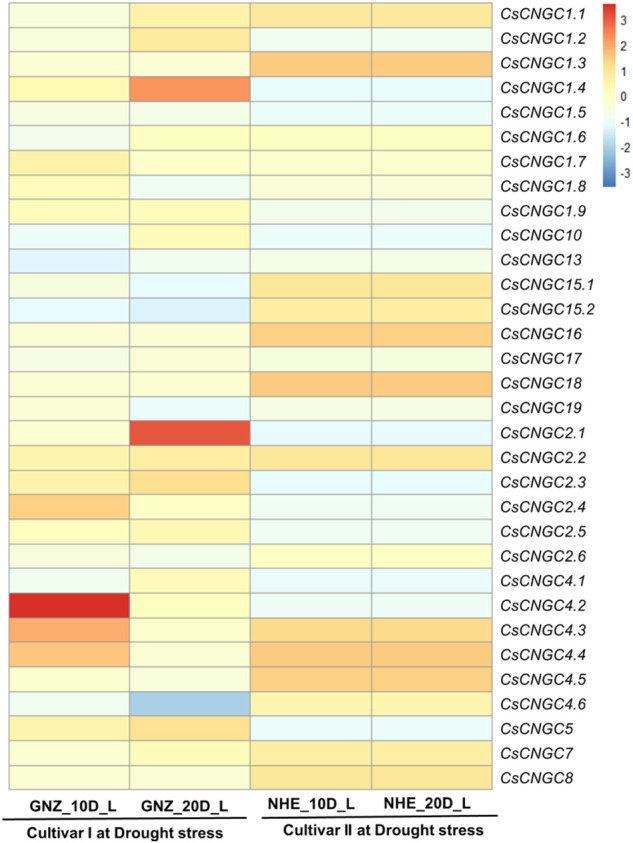
Heatmap representing the change in expression levels of *CsCNGC* genes in leaves under drought stress at 10 and 20 days. Red color represents up-regulation of *CsCNGCs*, Sky blue color represents downregulation of genes and the beige color represents no change in expression.

RNA-Seq data analysis was performed for leaves sample of *C. sinensis* under drought stress in two cultivars namely Newhall navel (NHE) orange, and Gannanzao (GNZ) navel orange at 0,10 and 20 days. The results suggest that *CsCNGC2.1* and *CsCNGC1.4* were highly up-regulated in cultivar I (20 days) and *CsCNGC4.2* (10 days). *CsCNGC1.3, CsCNGC15.1, CsCNGC15.2, CsCNGC16* and *CsCNGC18* were slightly up-regulated in cultivar II (Figure 8).

*CsCNGC1.1* was slightly up-regulated in cultivar I (20 days) and cultivar II. *CsCNGC2.2* was slightly up-regulated in both cultivars. *CsCNGC1.2* was slightly up-regulated in cultivar I (20 days). *CsCNGC1.7* was slightly up-regulated in cultivar I (10 days). *CsCNGC2.2* was slightly up-regulated in both cultivars. *CsCNGC2.3* was slightly up-regulated in cultivar I. *CsCNGC2.4* was slightly up-regulated in cultivar I (10 days). The increase in the expression level of these duplicated genes suggests that they not only evolved in number but in function also. *CsCNGC4.3 and CsCNGC4.4* were slightly up-regulated in cultivar I (10 days) and cultivar II. *CsCNGC4.5* was slightly up-regulated in cultivar II. *CsCNGC7* and *CsCNGC8* were slightly upregulated in cultivar II. *CsCsCNGC4.6* was highly down-regulated in cultivar I (20 days) and slightly down-regulated in cultivar I (10 days). *CsCNGC1.4, CsCNGC1.5, CsCNGC1.9, CsCNGC2.1, CsCNGC2.3, CsCNGC2.4, CsCNGC2.5, CsCNGC2.6, CsCNGC4.2* and *CsCNGC5* were slightly down-regulated in cultivar II. *CsCNGC1.8* was slightly down-regulated in cultivar I (20 days). Most of these genes have evolved through duplication which suggests that they are involved in the stress modulating process either by upregulating or downregulating their expression level. This change in their expression level may contribute to modulating stress response in drought stress as well. *CsCNGC10* was slightly down-regulated in cultivar I (10 days) and cultivar II. *CsCNGC13* was slightly down-regulated in both cultivars. *CsCNGC15.1, CsCNGC15.2, CsCNGC16* was slightly down-regulated in cultivar I. *CsCNGC19* was slightly downregulated in cultivar I (20 days). *CsCNGC1.1* and *CsCNGC1.2* in cultivar I (10 days), *CsCNGC1.3* and *CsCNGC1.5* in cultivar I, *CsCNGC1.6* in cultivar I (20 days) and cultivar II, *CsCNGC1.7* in cultivar I at (20 days) and cultivar II, *CsCNGC1.8* in cultivar I (10 days) and cultivar II, *CsCNGC1.9* in cultivar I, *CsCNGC10* in cultivar I (20 days), *CsCNGC17* in both cultivars, and *CsCNGC18* in cultivar I, *CsCNGC19* in cultivar I (10 days) and cultivar II, *CsCNGC2.1* in cultivar I (10 days), *CsCNGC2.4* in cultivar I (20 days), *CsCNGC2.6* in cultivar II, *CsCNGC4.1, CsCNGC4.2, CsCNGC4.3 and CsCNGC4.4* in cultivar I (20 days), *CsCNGC7* in cultivar I (10 days) and *CsCNGC8* in cultivar I were those genes that have no change in expression after providing stress condition (Figure 8).

### 3.10 Expression validation of the citrus cyclic nucleotide-gated channel genes through quantitative reverse transcription-polymerase chain reaction

**FIGURE 9:**
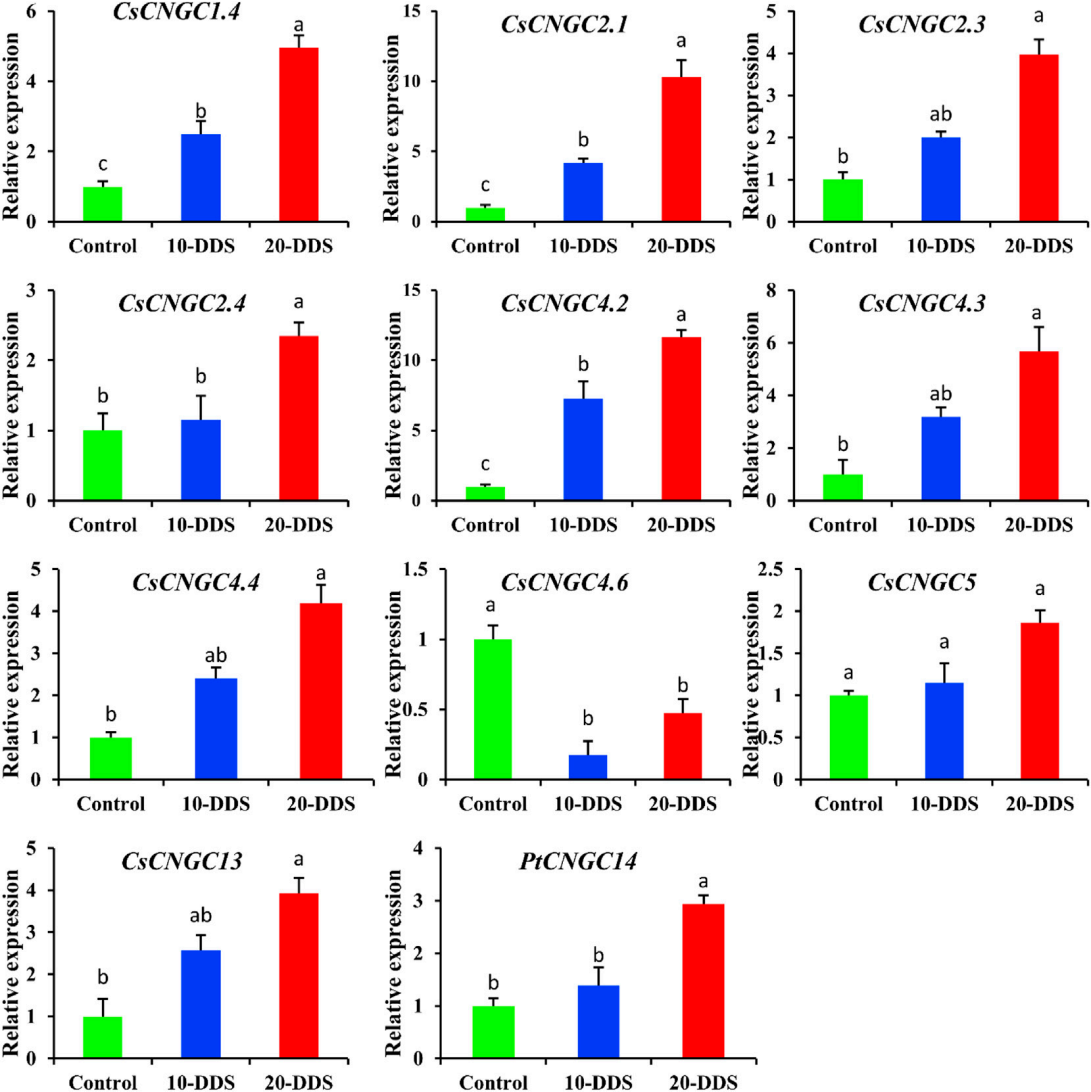
The graphs represent the qRT-PCR results of *CsCNGC* genes under drought stress. 10-DDS: 10 days of drought stress; 20-DDS: 20 days of drought. Each column represents the mean of three biological replicates. The least significant difference was applied to compare the difference between control and dissimilar drought stress levels at *p* < 0.05 (a, b, c).

To explore the role and relationship between *CNGC* genes and drought stress, the citrus plant was treated with drought stress under different conditions (Figure 9). The results showed the expression level of different genes under no treatment and drought treatment at 10 and 20 days. According to the qRT-PCR results, the *CsCNGC1.4* gene had higher expression after 10 and 20 days of drought treatment compared to the expression level when no stress was applied (Figure 9). The same pattern of gene expression was observed for other members including *CsCNGC2.1, CsCNGC2.3*, *CsCNGC2.4*, *CsCNGC4.2*, *CsCNGC4.3*, *CsCNGC4.4* and *CsCNGC5*. The level of gene expression increased after 10 days of treatment and further increased after 20 days of treatment. *CsCNGC4.6* had different expression patterns, where the level of gene expression under controlled conditions was higher. Drought treatment for 10 days decreased the level of gene expression, while the level of gene expression was again increased after 20 days of drought stress but still lesser than the controlled condition. Two unique genes *CsCNGC13* and *PtCNGC14* had the same expression pattern being lesser expression under controlled conditions while increased after treatment with drought stress. Results suggest that these members of the *CNGC* gene family were sensitive to stress conditions, and thus are involved in stress regulation.

### 3.11 3D Structure prediction of CNGCs in citrus spp.

**FIGURE 10:**
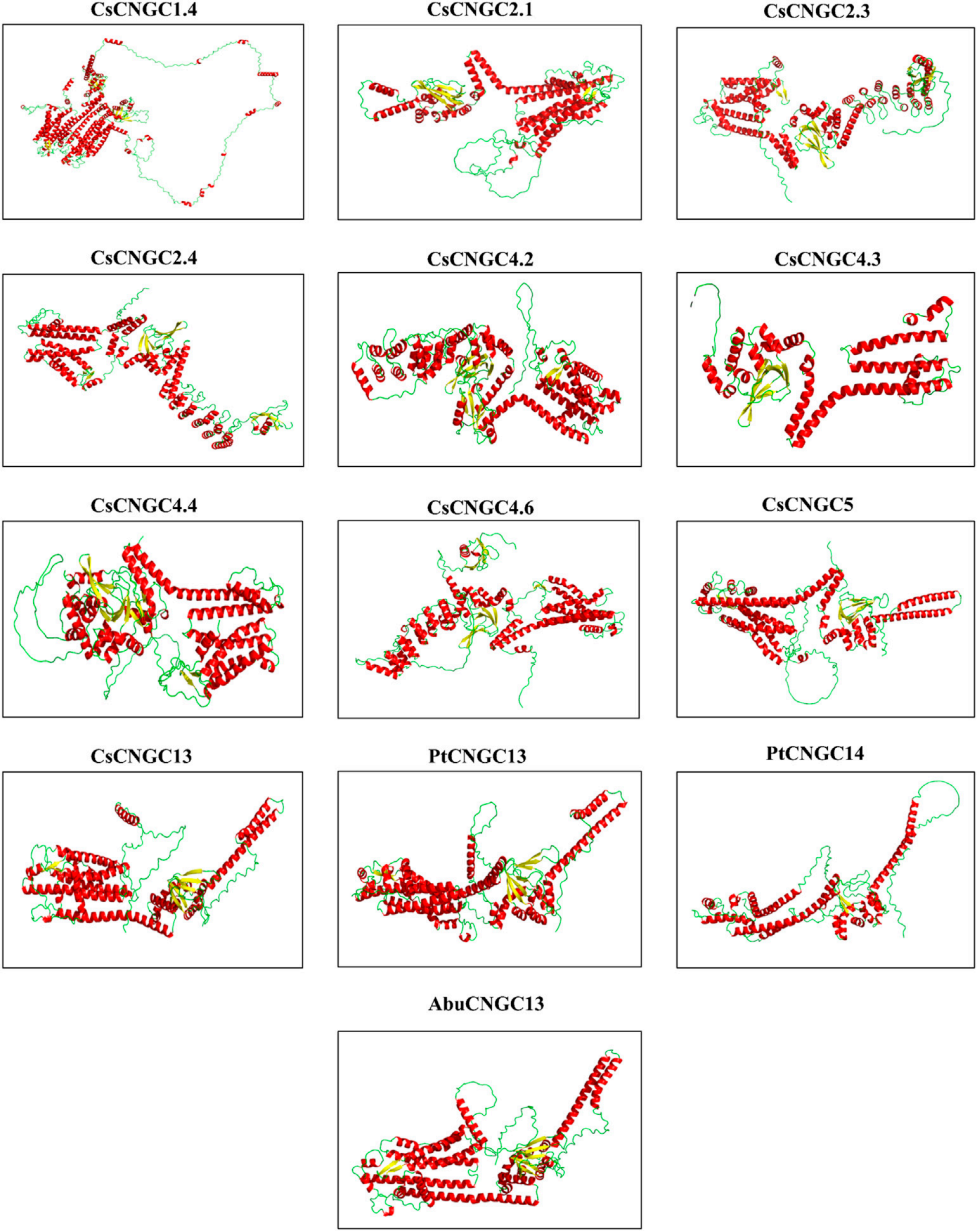
Predicted 3D structures of 12 CNGCs in *C. sinensis, A. buxfolia,* and *P. trifoliata* using Alphafold2. CsCNGC1.4 has been predicted by using tRrosetta. Structures are displayed based on secondary structures. Spirals with red color represent alpha helices, wide arrows with yellow color represent beta sheets, and wires with green color represent the loops.

The protein structures that were predicted are having almost similar structures except for CsCNGC1.4 and PtCNGC14 which had unique structures (Figure 10). Three-dimensional structures of solely thirteen CNGC proteins were predicted because these were differentially expressed proteins. Predicted structures of all CNGC proteins were visualized in the interactive 1 preset of Pymol (Yuan et al., 2017) where different colors are used to represent alpha helices and beta sheets. Each CNGC protein contained alpha helices and beta sheets. The long spirals were representing alpha helices while wide arrows were representing beta sheets. The templates used by tRrosetta for modeling the structure of CsCNGC1.4 were 5VA1, 7NP4, 5U6O, and 6UQF. CsCNGC1.4 had 55 alpha helices, CsCNGC2.3 had 38 alpha helices, CsCNGC2.4 had 37 alpha helices, and CsCNGC4.3 had 18 alpha helices, CsCNGC4.6 had 41 alpha helices, PtCNGC14 had 24 alpha helices and PtCNGC13 had 28 alpha helices. While CsCNGC2.1 and CsCNGC4.4 contained 27 alpha helices, CsCNGC13 and AbuCNGC13 contained 26 alpha helices. CsCNGC1.4 had 14 beta sheets, PtCNGC14 had 2 beta sheets, CsCNGC2.1, CsCNGC13, and AbuCNGC13 contained 8 beta sheets while the rest contained 10 beta sheets. The predicted structures of all these CNGC proteins were almost similar except for CsCNGC1.4 and PtCNGC14 suggesting that these proteins are potentially functionally similar too.

## 4 Discussion

The *CNGC* family is characterized by the presence of a CNBD domain and 6 TM domains along with a pore region (Saand et al., 2015). In the present study, the *CNGC* gene family is reported in *C. sinensis, C. recticulata, C. grandis, A. buxfolia, and P. trifoliata*. The presence of Ion trans and CNBD domain in *C. sinensis, C. recticulata, C. grandis, A. buxfolia,* and *P. trifoliata* confirm the genes identified are true *CNGC* genes. Most of the proteins in *B. oleracea* (Kakar et al., 2017)*, O. sativa* (Nawaz et al., 2014)*, Z. mays* (Hao and Qiao, 2018)*, T. aestivum* (Guo et al., 2018)*, Z. jujuba mill* (Wang et al., 2020). were basic, unstable, hydrophilic, and localized to the plasma membrane and similar results were found for citrus spp. in the present study. The localization of citrus CNGC proteins to the plasma membrane means that these are ion channel proteins and are involved in the uptake of calcium across the membrane. Pangenome-wide analysis provides a comprehensive overview of diversity at the genomic level involving multiple species, which may lead to the identification of unique genes which are present in specific species instead of being present in all genomes under study (Tahir ul Qamar et al., 2020). Similarly, in this study two unique genes were identified including *CNGC13* and *CNGC14.* The function of these members has not been yet identified in *A. thaliana*. Although, the function of these two members has been identified in *O. sativa* (Xu et al., 2017; Cui et al., 2020). The number of members in *C. grandis* and *P. trifoliata* is the same as the number of members in *B. rapa* while the number of members in Citrus *Spp.* is higher than that in *Z. mays* (12) (Hao and Qiao, 2018), *Z. jujuba* (15) (Wang et al., 2020), *O. sativa* (16) (Nawaz et al., 2014), *S. lycopersicum* (18) (Saand et al., 2015), *A. thaliana* (20) (Mäser et al., 2001), *P. bretschneideri* (21) (Chen et al., 2015), *B. oleracea* (26) (Kakar et al., 2017)*,* and lower than that in *N. tobacum* (35) (Nawaz et al., 2018), *T. aestivum* (47) (Guo et al., 2018). The phylogenetic analysis classified the *CNGC* family members into four major groups and two sub-groups, I, II, III, IV-A, and IV-B that were the same as *A. thaliana* but some members were missing in Citrus *Spp.* The members that belong to the same group could have similar structures and functions. Group members in *C. sinensis, C. recticulata, C. grandis, A. buxfolia, and P. trifoliata* were named by the phylogenetic relationships with *CNGC* members of *A. thaliana*. However, *CNGC1.1-1.5* and *CNGC10* were present in group I of Citrus *Spp.* While *CNGC13* which belongs to the same group was present only in *C. sinensis, A. buxfolia,* and *P. trifoliata*. *CNGC5, CNGC7,* and *CNGC8* were present in group II of Citrus *Spp. CNGC15.1-15.2, CNGC17,* and *CNGC18* were present in group III of Citrus *Spp.* While *CNGC14* belongs to the same group *and* was only present in *P. trifoliata, CNGC15.3* also belongs to the same group and was present in *C. recticulata, C. grandis, A. buxfolia,* and *P. trifoliata* except for *C. sinensis*. *CNGC16* also belongs to the same group and was present in *C. sinensis, C. recticulata, C. grandis*, and *P. trifoliata* while *CNGC16.1* and *CNGC16.2* were present in *A. buxfolia*. *CNGC19* was present in group IV-A of Citrus *Spp.,* while *CNGC2.1-2.3* and *CNGC4.1-4.6* were present in Group IV-B of Citrus *Spp. CNGC2.4, CNGC2.5,* and *CNGC2.6* also belong to the same group where *CNGC2.4* was present in *C. sinensis, C. recticulata, C. grandis,* and *P. trifoliata* except *A. buxfolia, CNGC2.5* and *CNGC2.6* were present in *C. sinensis, C. grandis* and *P. trifoliata* except *C. recticulata* and *A. buxfolia*. In the current study Group IV constituted the largest clade with 84 members while the clade of group II was the smallest with 29 members. While, in *A. thaliana* (Mäser et al., 2001) clade of group I was the largest and the clade of group IV was the smallest. In *B. rapa* (Li et al., 2019) group I constituted largest clade and clade of group IV-B was smallest. In *Z. mays* (Hao and Qiao, 2018) clade of group IV-B was largest and clade of group I was smallest. In *B. oleracea* (Kakar et al., 2017) clade of group IV was largest and clade of group II was smallest. In *P. bretschneideri* (Chen et al., 2015) clade of group I was largest and clade of group II and IV-A was smallest. In *O. sativa* clade of group III was largest and group II was smallest.

Results of chromosomal mapping suggested that most of the genes were present on chromosome 9 in *C. sinensis, C. recticulata, C. grandis, A. buxfolia,* and on chromosome 1 in *P. trifoliata.* Minimum genes were present in chromosomes 3, 6, and 8 in *C. sinensis,* chromosome 3 in *C. recticulata,* C*. grandis, and A. buxfolia* while chromosome 2 and chromosome 8 in *P. trifoliata.* The distribution of *CNGC* genes on chromosomes in Citrus *Spp.* was different as compared to other plants in which the gene family is already reported including *B. oleracea* (Kakar et al., 2017) in which maximum genes were present on chromosome 1 and 5 and minimum genes were present on chromosome 7. *B. rapa* (Li et al., 2019) in which maximum genes were present on chromosome 1 and minimum genes were present on chromosomes 6, 7, and 9, *P. bretschneideri* (Chen et al., 2015) in which maximum genes were present on chromosomes 1, 8, and 15, and minimum genes were present at 2, 9, 13, 16 and 17, *N. tobaccum* (Saand et al., 2015) in which chromosome 1 and 8 carried maximum genes and minimum genes were present at chromosome 22 and 11. The gene structures of *C. sinensis, C. recticulata, C. grandis, A. buxfolia, and P. trifoliata* were somewhat similar to *A. thaliana* as the number of exons and introns of Citrus plants that are being studied were not exactly same as *A. thaliana*, *O. sativa,* and other plants. Conserved motif analysis suggested that motifs for IQ domain, CaM binding motif, and CNB motifs were present in *C. sinensis, C. recticulata, C. grandis, A. buxfolia, P. trifoliata* as reported in *B. oleracea* (Kakar et al., 2017) in which all the above-mentioned motifs were present. *Z. jujube* (Wang et al., 2020) also had a similar pattern of motifs. Others include *N. tobaccum* (Nawaz et al., 2018) in which CNB motif CaM binding motif and motif for IQ domain were present, and *T. aestivum* (Guo et al., 2018) in which Cyclic nucleotide binding motif and motif for IQ domain were present. In *Z. mays* (Hao and Qiao, 2018) motif 3 was the combination of both CaMB and motif for the IQ domain, while motif 4 was the CNB domain and motifs 1, 2, 5, 8, 9, and 10 were transmembrane domains. The motifs were closely related to CNGC motifs in *Z. mays*. Cis-regulatory elements (CREs) that were present in promoter regions of Citrus *Spp.* were mainly of four types light responsive, stress-related, hormone-related, and development related. In *Z. mays* (Hao and Qiao, 2018) hormones, stress, and development-related cis-regulatory elements were present. *O. sativa* (Nawaz et al., 2014)*, Z. jujuba* (Wang et al., 2020)*,* and *N. tobaccum* (Nawaz et al., 2018) also contained all these stress-responsive elements. Cis-regulatory element analysis shows that the *CNGC* gene family is involved in plant response to light, hormone, and abiotic Gene duplication mainly contributes to the expansion of a gene family in plant species. In *P. bretschneideri* mainly segmental duplication has played role in the expansion of the *CNGC* gene family (Chen et al., 2015). In Arabidopsis *CNGCs* both segmental and tandem duplications contributed to the expansion of the CNGC gene family. Similarly, both segmental and tandem duplications played a role in the expansion of the *CNGC* gene family in *C. sinensis, C. recticulata, C. grandis, A. buxfolia,* and *P. trifoliata*. In *O. sativa* (Nawaz et al., 2014) three gene pairs were found to be segmentally duplicated including *OsCNGC1/OsCNGC2, OsCNGC10/OsCNGC11, OsCNGC15/OsCNGC16,* and one gene pair was found to be tandemly duplicated including *OsCNGC2/OsCNGC3*. Hence, both tandem and segmental duplications contributed to the expansion of the *CNGC* gene family in *O. sativa* (Nawaz et al., 2014). In *N. tobacum* the *CNGC* gene family was also considered to be expanded through both segmental and tandem duplications (Nawaz et al., 2018). Most of the *OsCNGCs* were upregulated under abscisic acid treatment (ABA) i.e., 12 and indole acetic acid (IAA) treatment i.e., 11, and very few genes were upregulated under kinetin (KN) i.e., 2 and ethylene (ETH) treatment i.e., 6, where genes belonging to same groups showed similar expression patterns. Under cold stress *OsCNGCs* that were present in phylogenetic groups I, II, and III were upregulated and those present in group IV were downregulated where *OsCNGC6* exhibited the highest expression and *OsCNGC16* exhibited the lowest expression. Under pathogen stress where two phytopathogens were inoculated with 4 weeks old rice seedlings including *Pseudomonas fuscovaginae* and *Xanthomonas oryzae pv. oryzae (Xoo)* the expression patterns of *OsCNGCs* demonstrated that except *OsCNGC5* and *OsCNGC6* all other *OsCNGCs* were up-regulated under *Xoo* while all the fourteen *OsCNGCs* were significantly up-regulated under *P. fuscovagine* inoculation. Thus, all the OsCNGCs that were duplicated were exhibiting similar expression patterns alongside relevance in their functions. *OsCNGC1* and *OsCNGC2* were duplicated genes and were also exhibiting similar expression patterns under abiotic stress i.e., Abscisic acid (ABA) and indole acetic acid (IAA) treatment and pathogenic stress that demonstrates that their functions were overlapping (Nawaz et al., 2014). The 10 duplicated gene pairs in *C. sinensis* exhibit similar expression patterns except *CsCNGC2.1/CsCNGC2.2* where *CsCNGC2.1* was highly up-regulated in cultivar I at 20 days drought stress and slightly down-regulated in cultivar II while that as not true for *CsCNGC2.2*. Among 10 duplicated gene pairs *CsCNGC1.8* was slightly up-regulated in cultivar I at 20 days drought stress, *CsCNGC1.9* was slightly down-regulated in cultivar II, *CsCNGC2.3*, *CsCNGC2.4* and *CsCNGC2.5* were slightly down-regulated in cultivar II, *CsCNGC2.4* was slightly up-regulated in cultivar I at 10 days drought stress, *CsCNGC2.6* was slightly down-regulated in cultivar I at 20 days drought stress while *CsCNGC7* and *CsCNGC8* in both cultivars and aforementioned genes in remaining cultivars were having no change in expression. Thus, we can hypothesize that duplicated genes exhibit similar expression patterns and function overlapping in Citrus *Spp.* too. It seems that some evolutionary events such as duplication could affect the members of CNGC gene family. On the other hand, mutations in the structure, including upstream/downstream site and coding sequence site of members could change the expression levels of CNGC genes (Abdullah et al., 2021; Faraji et al., 2021; Heidari et al., 2021). In *T. aestivum* (Guo et al., 2018), *O. sativa* (Nawaz et al., 2014)*, A. thaliana* (Mäser et al., 2001)*, P. bretschneideri* (Chen et al., 2015)*, Z. mays* (Hao and Qiao, 2018)*, Z. jujuba* (Wang et al., 2020)*,* and *S. lycopersicum* (Saand et al., 2015) the CNGC family members were different indicating that gene duplications and gene losses have played an important role in the creation of new genes and functions. The increase in the number of *CNGC* gene family members was an important event that contributed to the ability of these plants to adapt to changing environmental conditions.

The miRNAs are non-coding RNAs that regulate gene expression. In this study, a total of 226 putative miRNAs were identified that targeted 32 *CsCNGCs*. Several miRNAs were targeting each gene except *CsCNGC17* which was targeted by a single miRNA and *CsCNGC7* was targeted by 16 miRNAs. In *B. oleracea* 14 miRNAs were identified that targeted 17 *BoCNGCs* (Kakar et al., 2017)*.* After eliminating false positives based on a threshold value of 5 there remained 5 miRNAs that targeted 9 *BoCNGCs.* Out of these miRNAs, bol-miR838days had five target genes while the rest of them were targeting only one gene. The majority of the miRNAs were related to cleavage while only two miRNAs were involved in the inhibition of translation of target genes. In *N. tobacum* 162 tobacco miRNAs were identified that targeted 18 *NtabCNGCs* (Nawaz et al., 2018)*.* After eliminating false positives based on a threshold value of 4 there remained 79 miRNAs. While, after applying a threshold value of 3 there remained 6 miRNAs from 3 families that comprised 8 *NtabCNGCs*. Most of the genes were having target sites for multiple miRNAs except *NtabCNGC19* which contained the target site of a single miRNA. Prior studies support the evidence that miRNAs are involved in stress response and adaptation including topping and wounding in *N. tobacum* and miRNAs are also involved in drought signaling in rice (Root, 2016). The study done by (AAB et al., 2019) demonstrates a list of drought-tolerant plant crops with the involvement of genes of specific gene families and the role of their respective miRNAs. Hence, we can conclude that miRNAs in CsCNGCs will also be involved in their response to drought stress. PPI network analysis showed the interaction among citrus CNGC proteins as well as with the other citrus proteins. Higher connectivity was shown by CNGC and other genes which shows their involvement in pathways. The PPI results performed on BoCNGC proteins show that these proteins also have higher connectivity among themselves and with other proteins suggesting their integrated role in biotic, abiotic stress, and hyper-sensitivity resistance (Kakar et al., 2017). In maize, the PPI network analysis was conducted based on interactions found on STRING. Similarly, the ZmCNGC proteins also showed connectivity within the CNGC members as well as with the homologous proteins from *Arabidopsis* (Hao and Qiao, 2018). In cotton, the functional interaction analysis demonstrated that most of the GhCNGC proteins were found to have higher connectivity with a receptor kinase present in the plasma membrane, FLS2 that activates immune signaling. Several other proteins were showing interactions with RSTK, MOL, and TAD3 which are involved in growth and developmental functions (Zhao et al., 2022). These results regarding interactions of CNGC family members show their contribution of these genes to the functional as well as regulatory diversity in plants and might be helpful in future research to better understand the functions of *CNGC* genes. As CNGCs are ion channels, so according to GO enrichment these genes are present in the plasma membrane, act as transmembrane ion transporters, and are involved in ion channel activity, potassium and calcium ion transport activity, and protein binding activity. In *Brassica oleracea*, according to biological processes, the BoCNGCs are associated with ion channel activity for transmembrane transport, negative regulation of defense responses, salicylic acid biosynthesis, responses to chitin, and plant-type hypersensitive responses. BoCNGCs are present in the plasma membrane and participate in cellular activities related to transduction, binding, and transport (Kakar et al., 2017).

Expression patterns of *CsCNGC* in leaves samples under drought stress at 10 and 20 days indicated that three genes namely *CsCNGC1.4, CsCNGC2.1,* and *CsCNGC4.2* were highly up-regulated while *CsCNGC4.6* was highly down-regulated. Out of two unique genes identified in this study, one is present in *C. sinensis*, *CsCNGC13.* The expression analysis of this gene in two cultivars is down-regulated under drought stress which shows some specialty in terms of abiotic stress regulation. These results were similar to the ones demonstrated by earlier studies such as expression patterns of *N. tobaccum* showed that 18 *CNGC* genes (*NtabCNGC2, 3, 5–7, 14, 16–21,* and *29–34*) were up-regulated under Calmodulin stress, 16 *CNGC* genes (*NtabCNGC1, 3–7, 14, 16, 17, 26–28,* and *30–33*) under drought stress and 10 *CNGC* genes (*NtabCNGC2, 3, 5–7, 14, 16, 17, 19* and *20*) under cold stress and some genes were downregulated in response to these stresses (Nawaz et al., 2018). Expression patterns of *O. sativa* demonstrated that 10 *OsCNGC* genes were up-regulated under cold stress, and group IV members were down-regulated under cold stress (Nawaz et al., 2014). In *Z. jujuba ZjCNGC10,* 8*,* 2*,* and 15 were downregulated under cold stress (24 h), and *ZjCNGC4* and 12 were up-regulated under cold stress (1 h). The majority of *ZjCNGCs* were down-regulated after being treated with salt stress, particularly group III members, and the same was the case for *ZjCNGCs* under alkaline stress (Wang et al., 2020). In *B. oleracea* 13 *BoCNGCs* genes were up-regulated under cold stress. However, more *BoCNGCs* were up-regulated under pathogen stress of *Xanthomonas campestris pv. campestris* (Xcc) as compared to those treated with cold stress (Kakar et al., 2017)*.* Promoter and expression analysis revealed some genes that have variable expression under abiotic stress. It is hypothesized that several hormones and abiotic stress-related elements control the variable expression level of *CsCNGCs* under various abiotic stress conditions. As a result, this study confers that these genes can be used in future research due to their importance in abiotic stress response.

## 5 Conclusion

In this study, a total of 32 genes in *C. sinensis*, 27 genes in *C. recticulata*, 30 genes in *C. grandis*, 31 genes in *A. buxfolia,* and 30 in *P. trifoliata* were identified as belonging to the *CNGCs* gene family. *CNGC* genes were identified based on CNGC-specific motifs and domains. *CsCNGCs, CreCNGCs, CgCNGCs, AbuCNGCs, and PtCNGCs* have diversity in their functions, protein lengths, and gene structures. Previously, Genome-wide studies have been done on the *CNGC* gene family in other plants but the present study is illustratating a pangenome-wide representation of the *CNGC* gene family among five Citrus *Spp*. To the best of our knowledge, this is the first research implementing the concept of pangenome-wide analysis and will be helpful for further pan-genome wide studies on other plants in the future. This analysis provided a detailed explanation regarding the pattern of evolution of *CNGCs* in Citrus *Spp.* their intron-exon patterns, distribution of *CNGC* genes on chromosomes, prediction of *CNGC* specific motifs and domains, duplication type, along with promoter region analysis indicating which regulatory elements are more likely to influence the expression of particular genes. Phylogenetic analysis revealed that *CNGCs* of these five citrus species were clustered into four major groups and two sub-groups. A few *CNGCs* in the groups were missing or might be duplicated during evolution. CREs analysis reveals the association of gene families in response to abiotic stresses. The miRNAs also play a role in the response of *CNGC* genes to drought stress alongside regulating the expression of these genes. PPI network analysis also provided insights into their connectivity suggesting their involvement in functional regulation. GO enrichment was executed to understand the functions of *CNGCs* at the molecular level. Expression profiling was done on tissue-specific data of *C. sinensis* under drought stress that demonstrates that *CsCNGC1.4*, *CsCNGC2.1*, *CsCNGC4.2* were highly upregulated and *CsCNGC4.6* was highly downregulated under drought stress. Unique genes *CsCNGC13* and *PtCNGC14* also showed higher expression in drought stress. These genes can be used in further studies to develop stress-resistant crops. One can visualize and understand the genomic diversity among the Citrus species being examined. We have observed significant inter and intra-species diversity of the CNGC gene family members. The diversity observed could be due to differences in sequencing approaches. Therefore, further experiemnts are required to get deep insights.

## Data Availability

The original contributions presented in the study are included in the article/Supplementary Material, further inquiries can be directed to the corresponding author.
