# Supplementary material for: Pangenome-wide analysis of cyclic nucleotide-gated channel (CNGC) gene family in citrus Spp. Revealed their intraspecies diversity and potential roles in abiotic stress tolerance
Source: Front Genet. 2022 Oct 11;13:1034921. doi: 10.3389/fgene.2022.1034921 (PMC9593079; doi:10.3389/fgene.2022.1034921)
Supplement: Supplementary file 1 [file DataSheet1.ZIP › Suppl. Figures.docx]

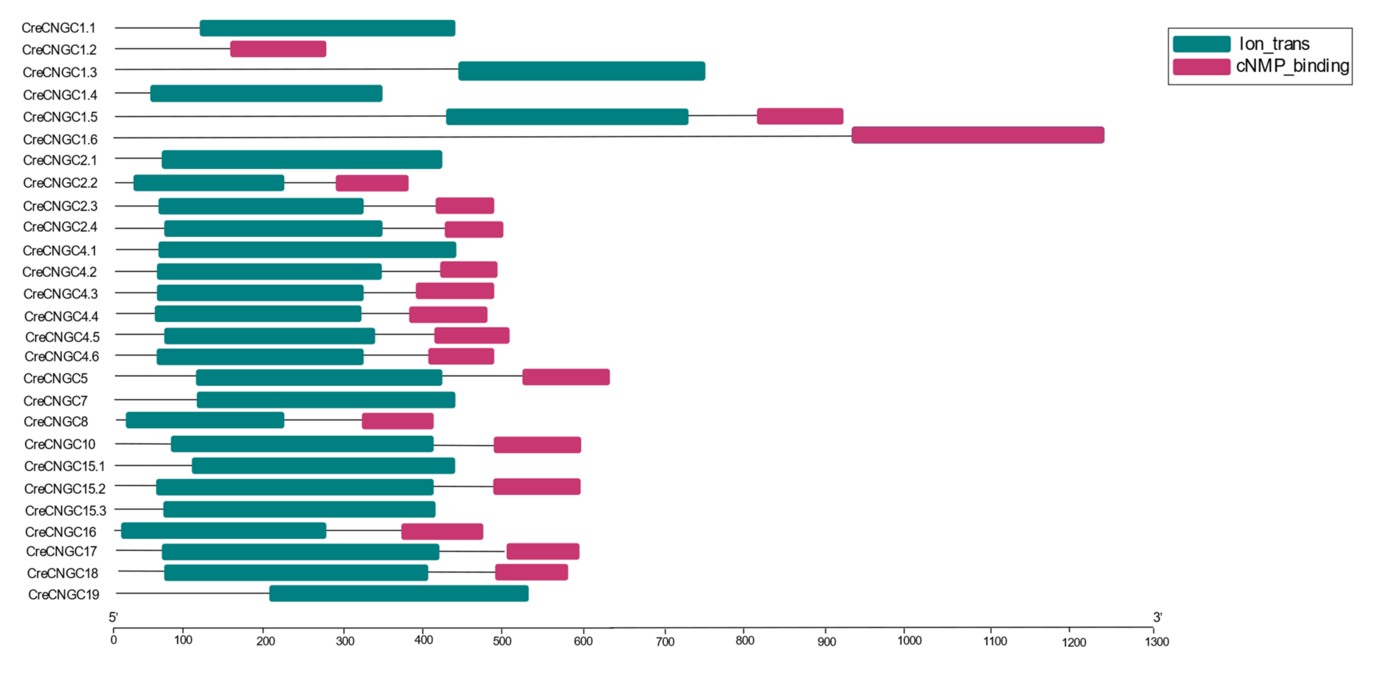


**Fig S1:** Domain architecture of CreCNGCs.


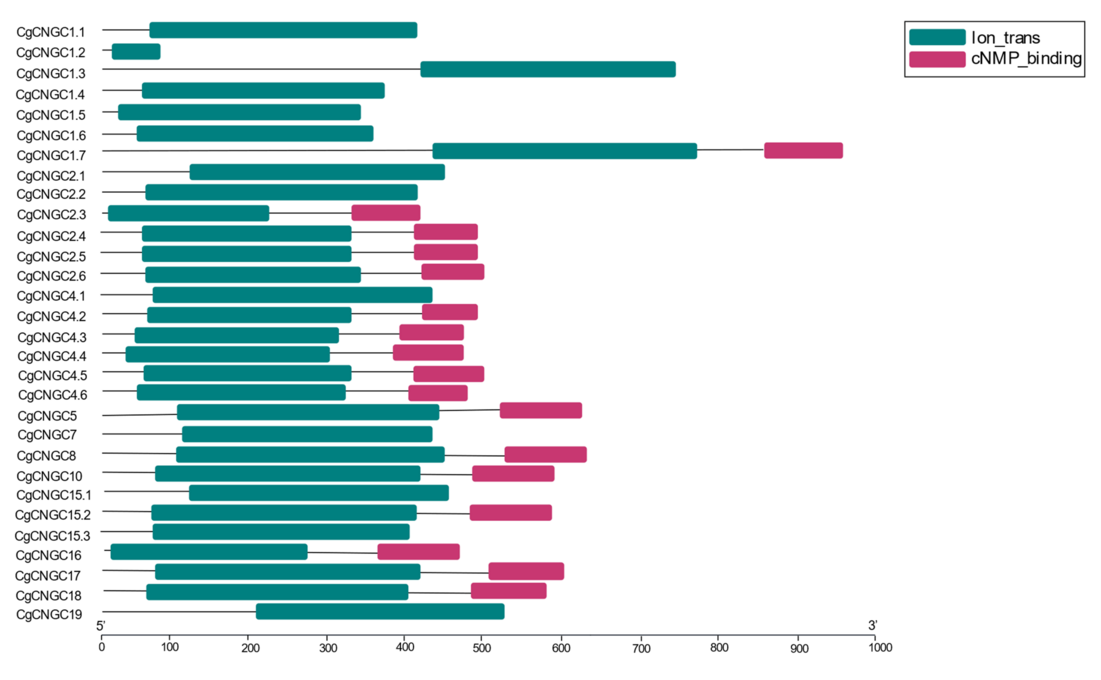


**Fig S2:** Domain architecture of CgCNGCs.


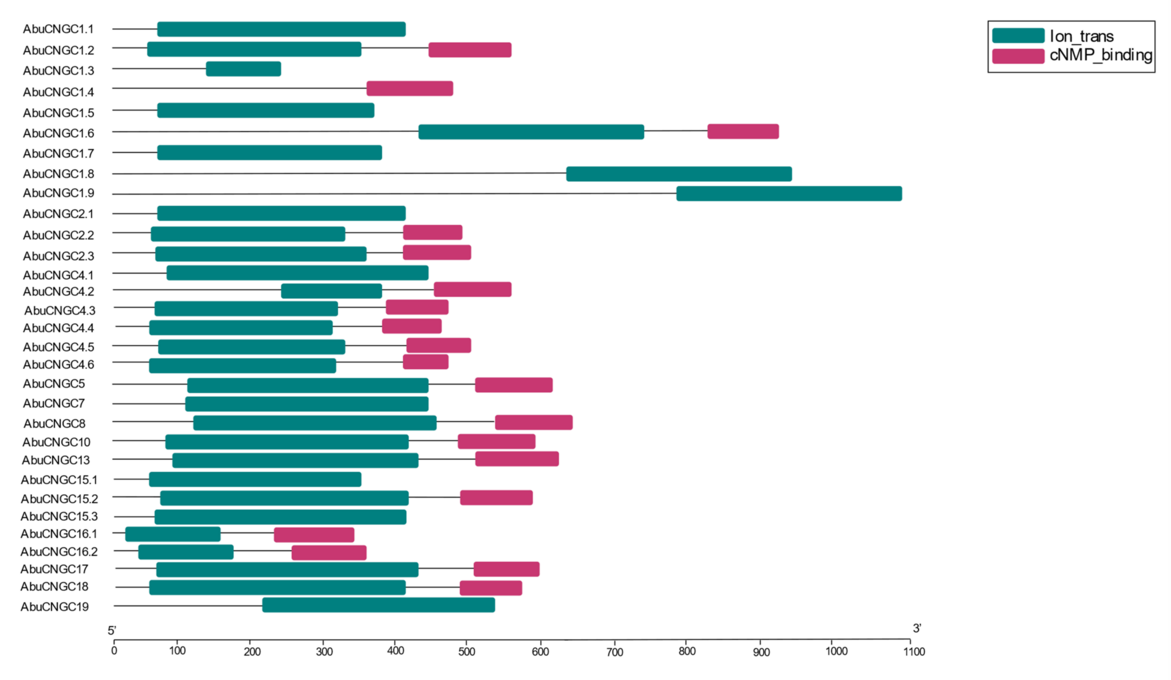


**Fig S3**: Domain architecture of AbuCNGCs.


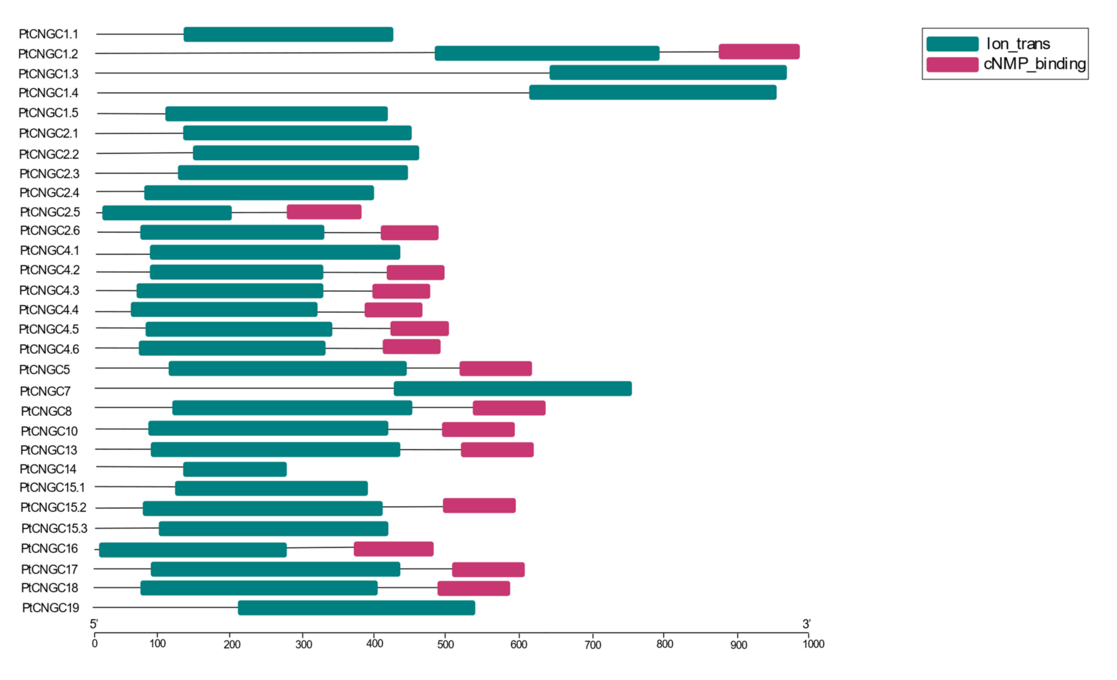


**Fig S4**: Domain architecture of PtCNGCs.


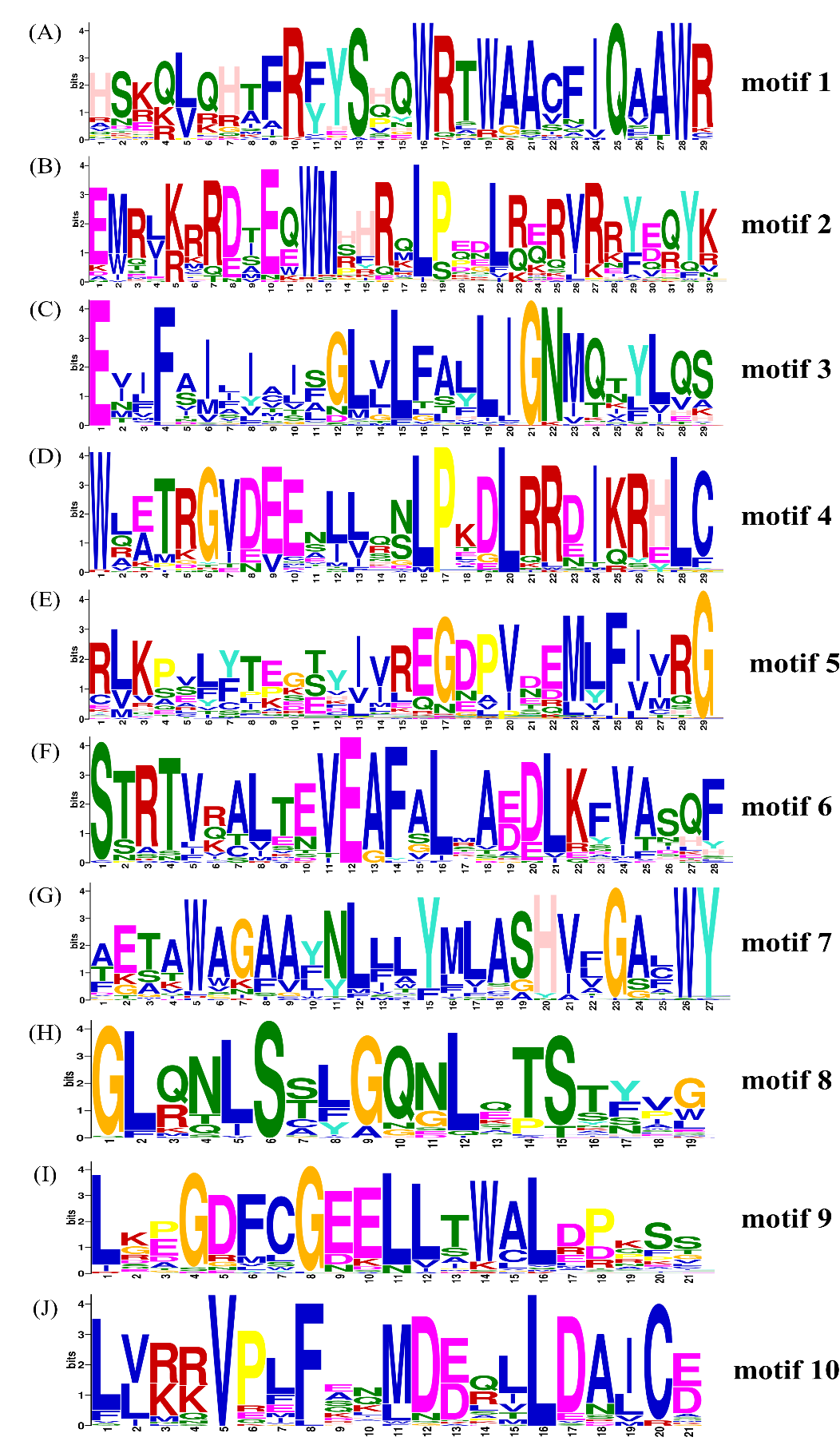


**Fig S5**: Logo of 10 conserved motif *C. sinensis.*


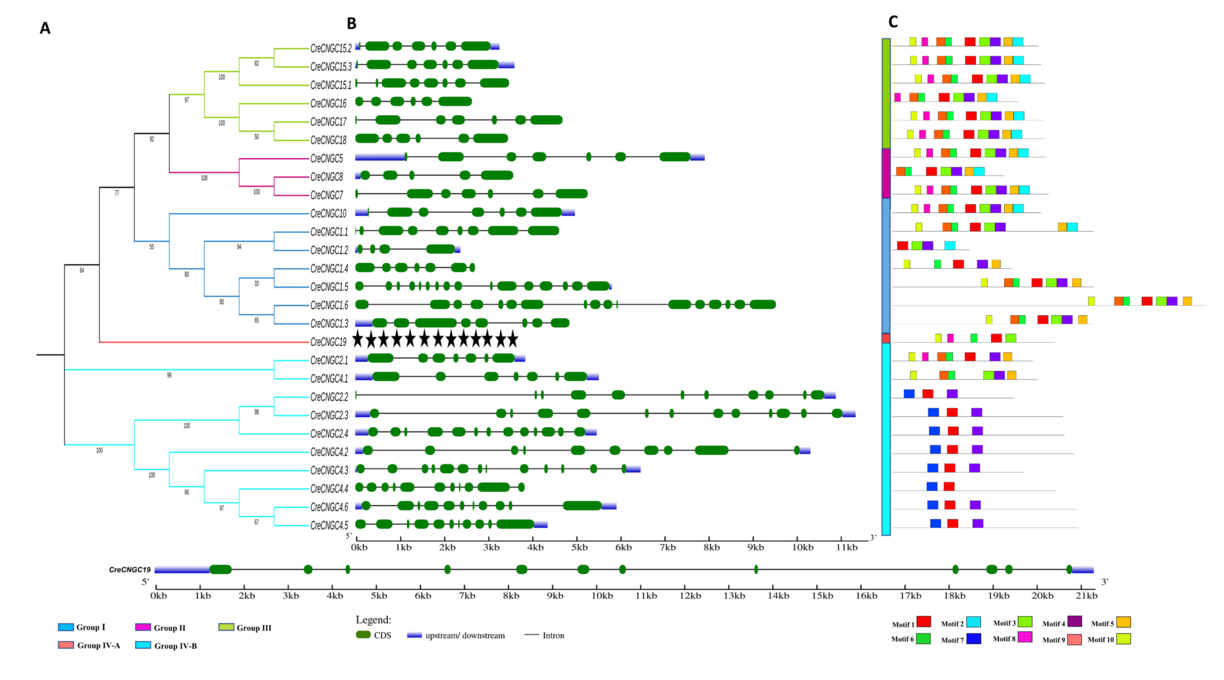


**Fig S6A**: Gene structure and motif analysis of CreCNGC.


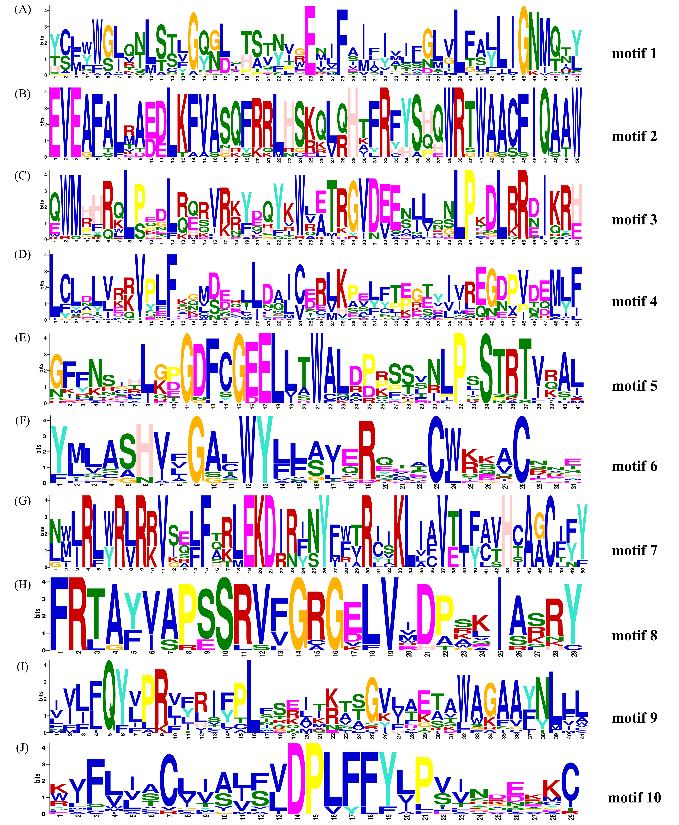


**Fig S6B**: Logo of 10 conserved motifs of CreCNGC.


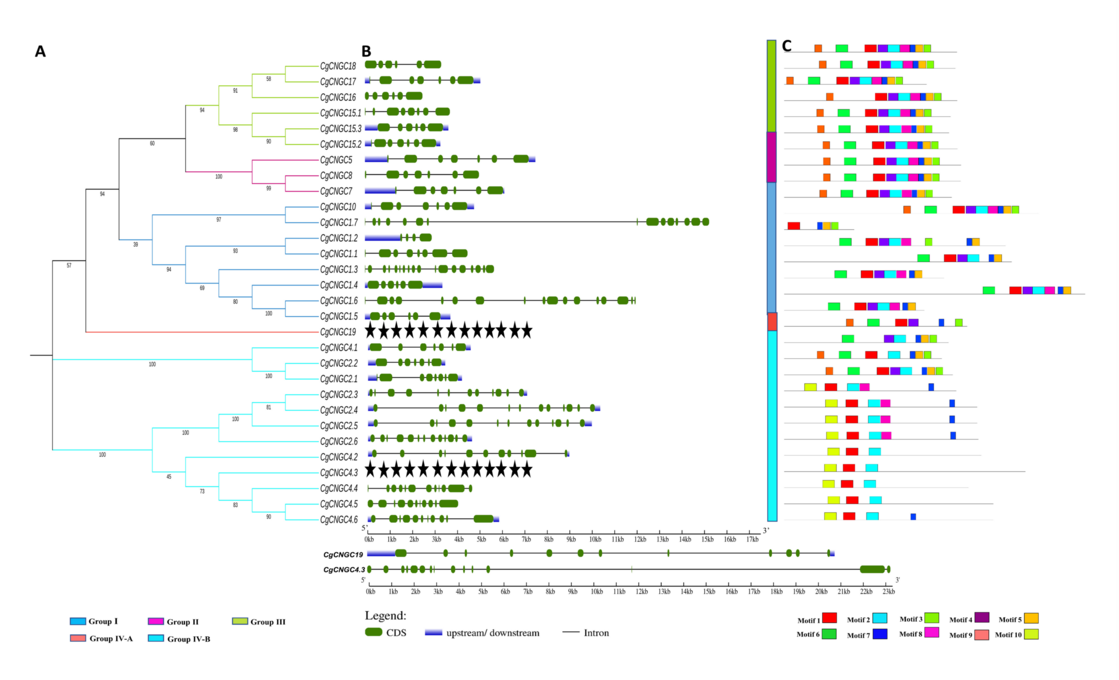


**Fig S7A**: Gene structure and motif analysis of CgCNGCs.


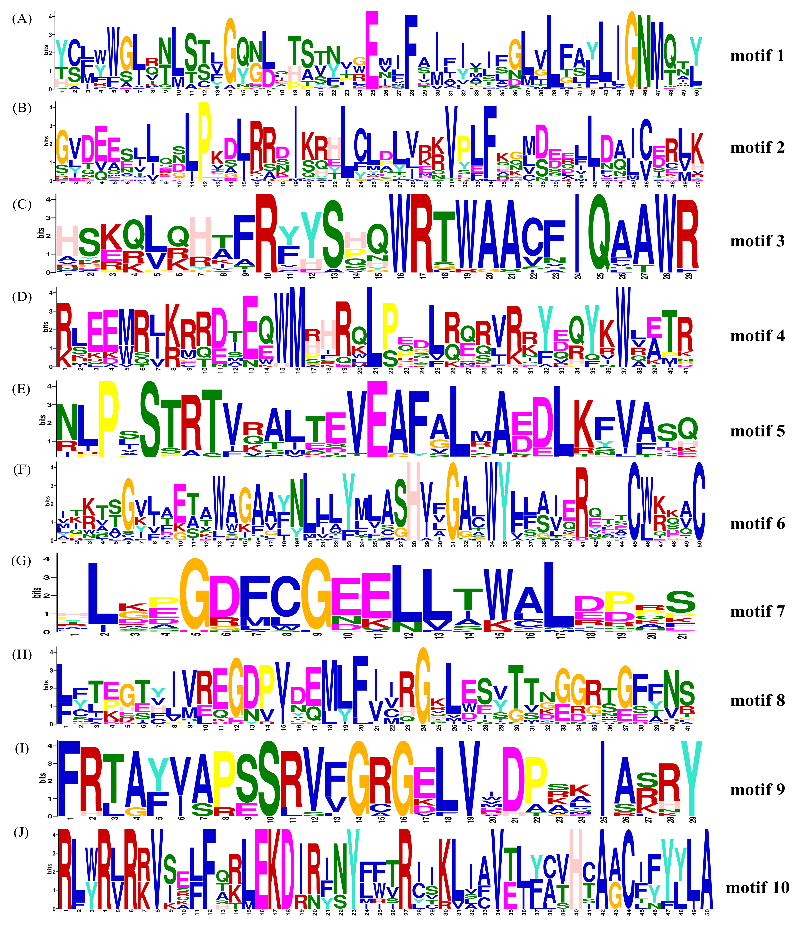


**Fig S7B**: Logo of 10 conserved motifs of CgCNGCs.


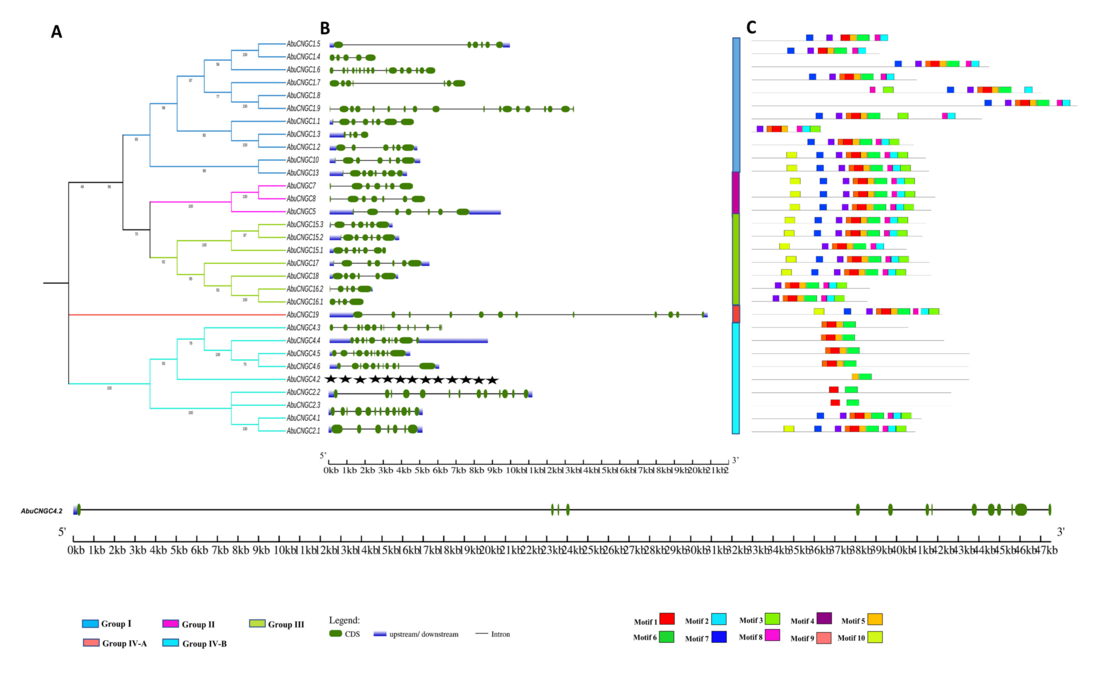


**Fig S8A**: Gene structure and motif analysis of AbuCNGCs.


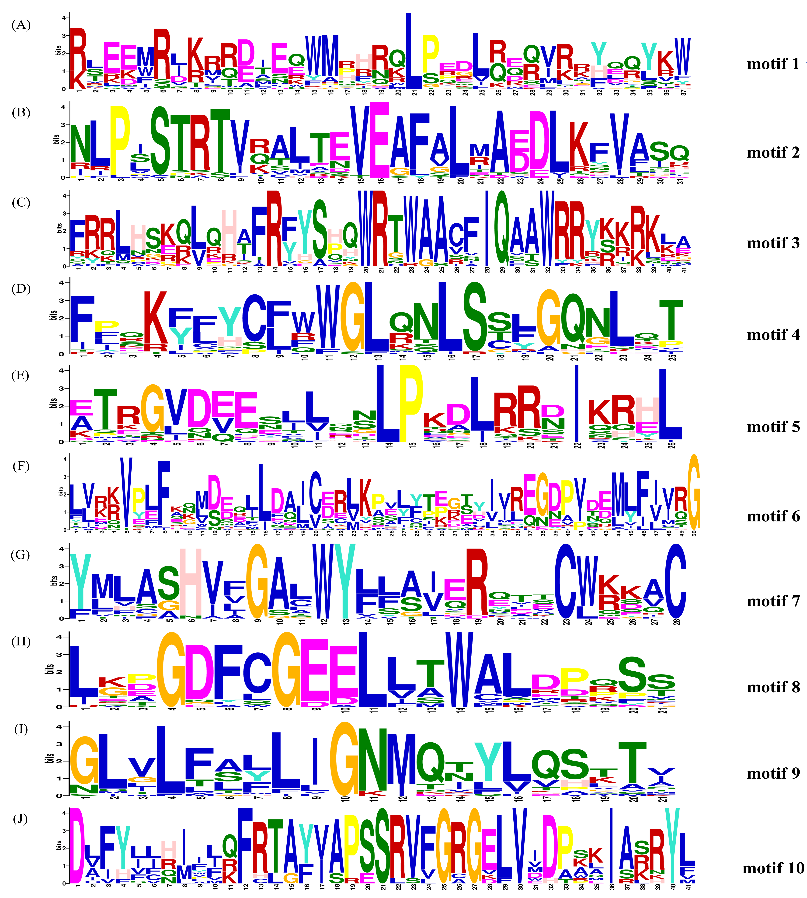


**Fig S8B**: Logo of 10 conserved motifs of AbuCNGCs.


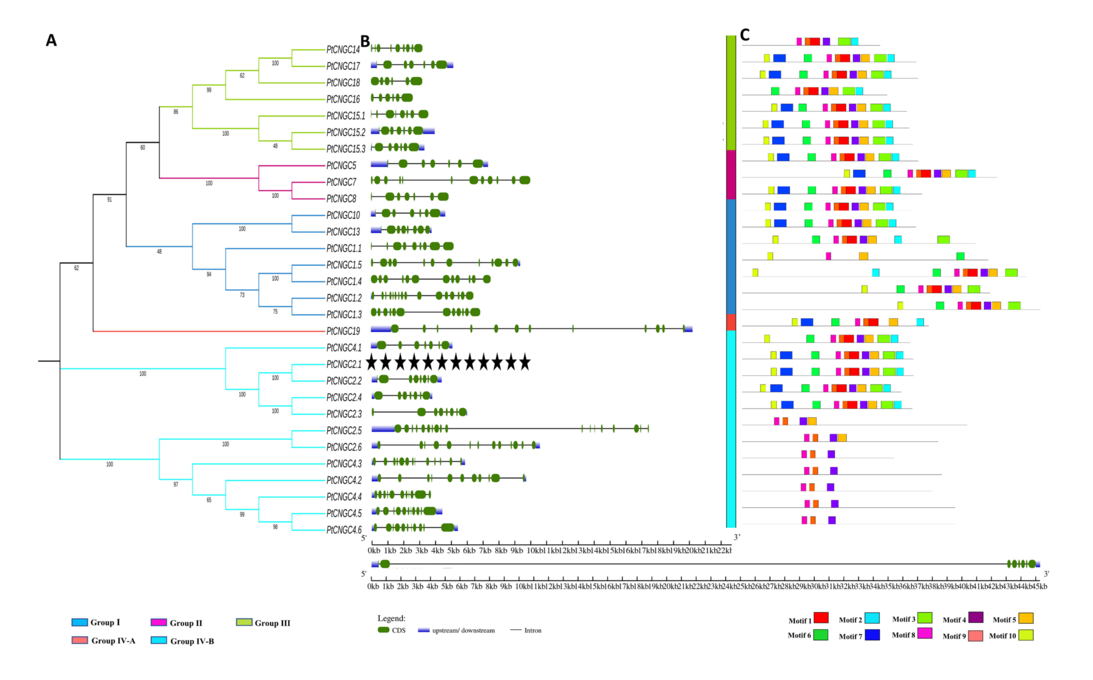


**Fig S9A**: Gene structure and motif analysis of PtCNGCs.


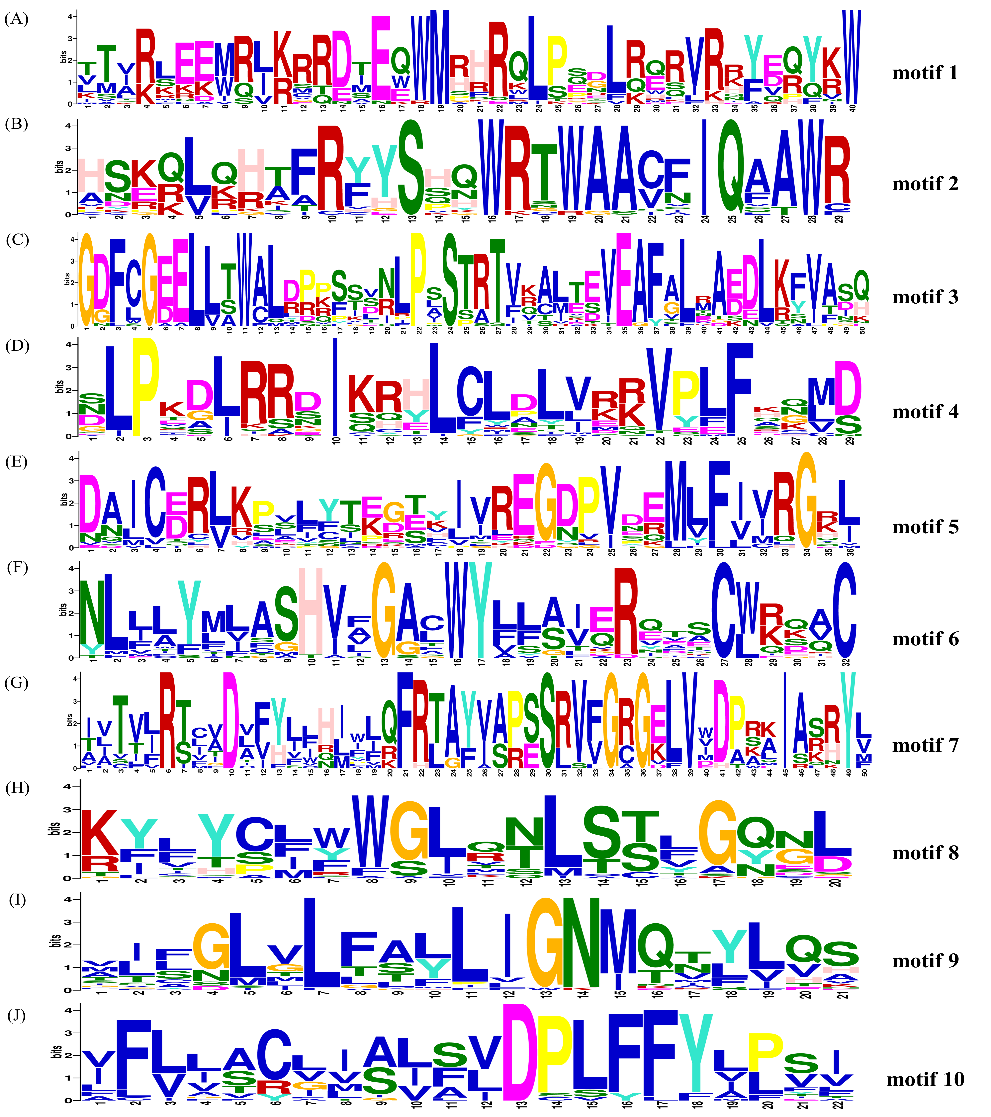


**Fig S9B**: Logo of 10 conserved motifs of PtCNGCs.


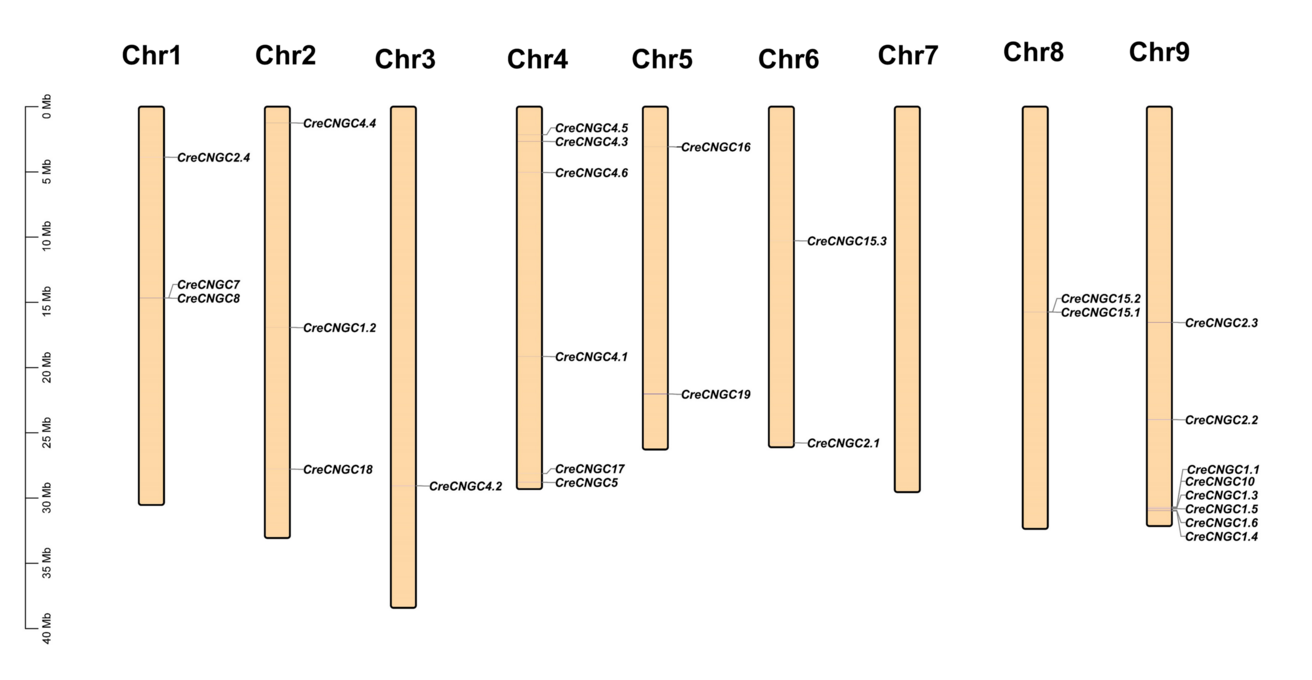


**Fig S10**: Chromosomal mapping of CreCNGC.


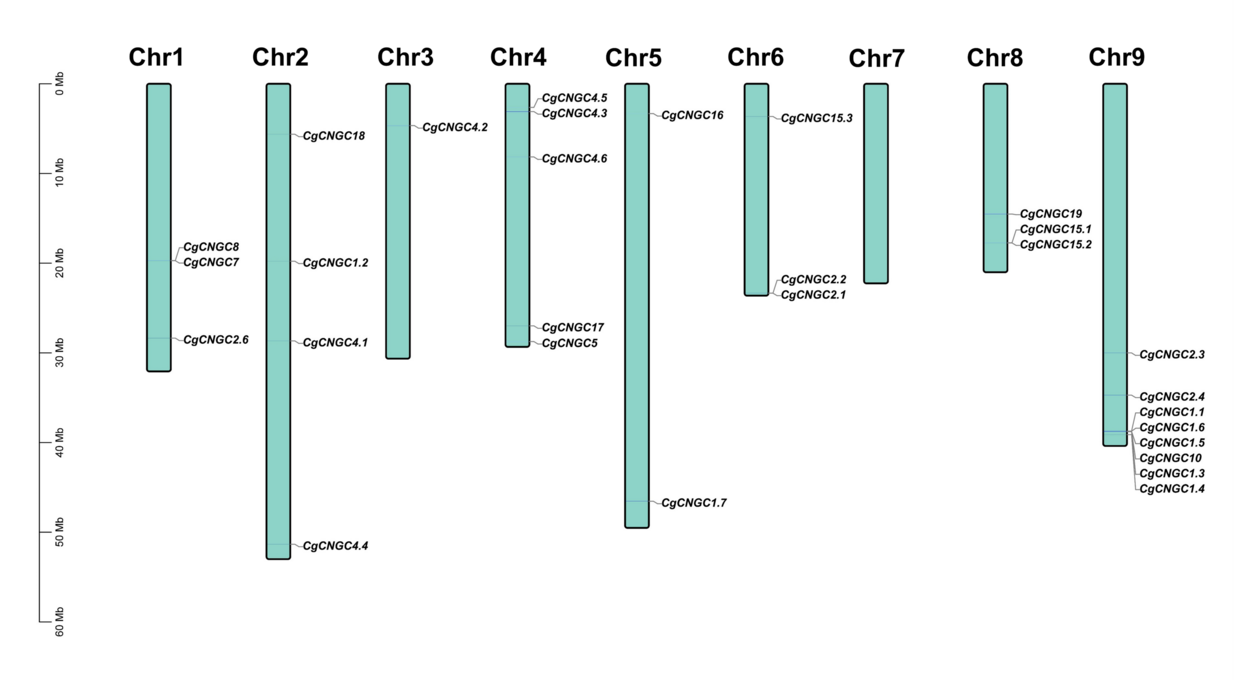


**Fig S11**: Chromosomal mapping of CgCNGCs.


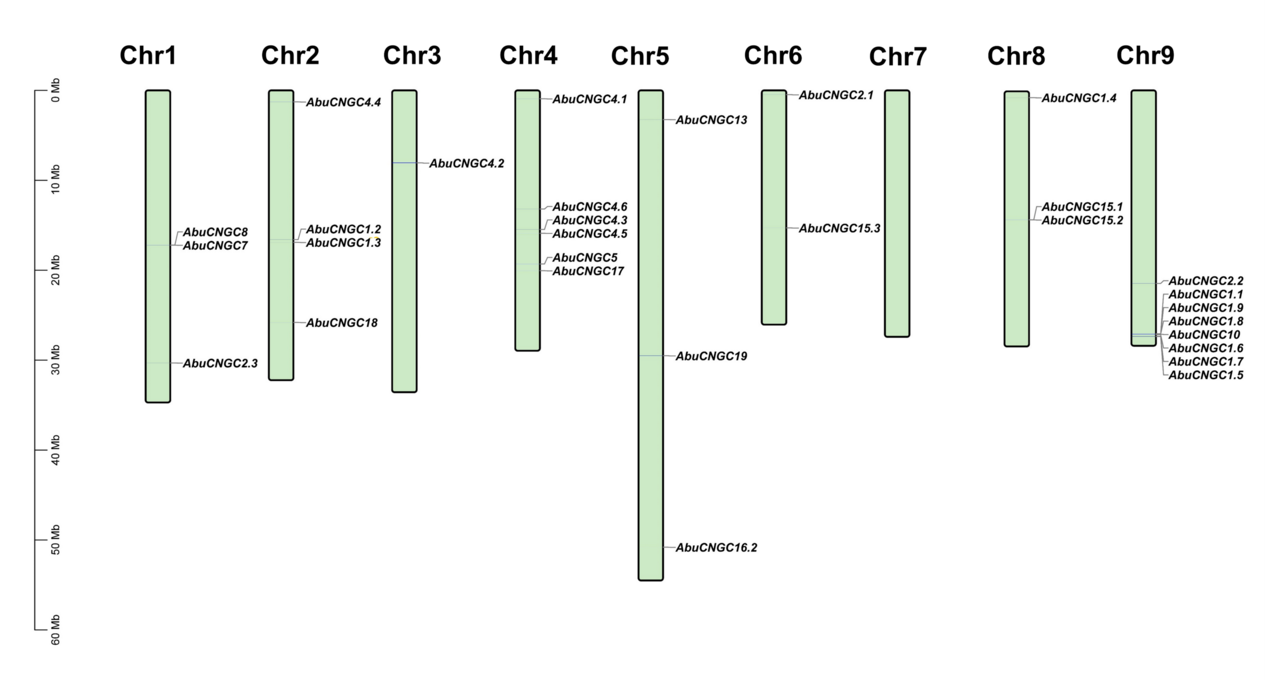


**Fig S12**: Chromosomal l mapping of AbuCNGCs.


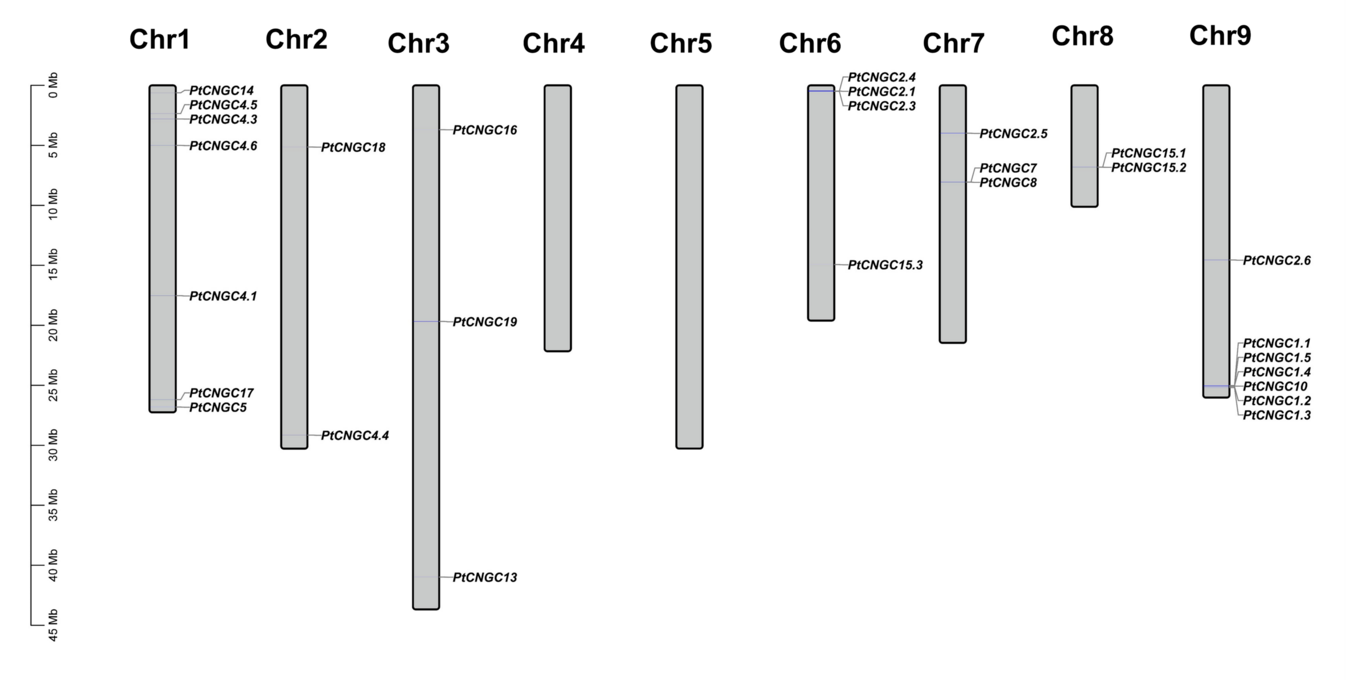


**Fig S13**: Chromosomal mapping of PtCNGCs.


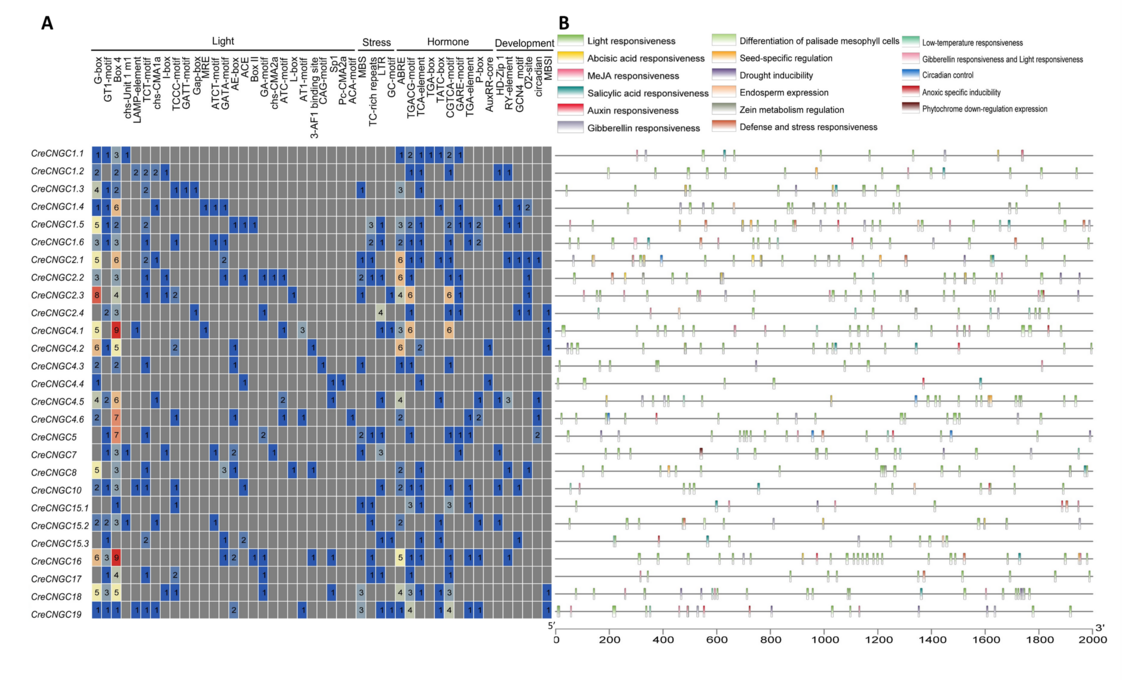


**Fig S14**: Cis-regulatory elements analysis of CreCNGC.


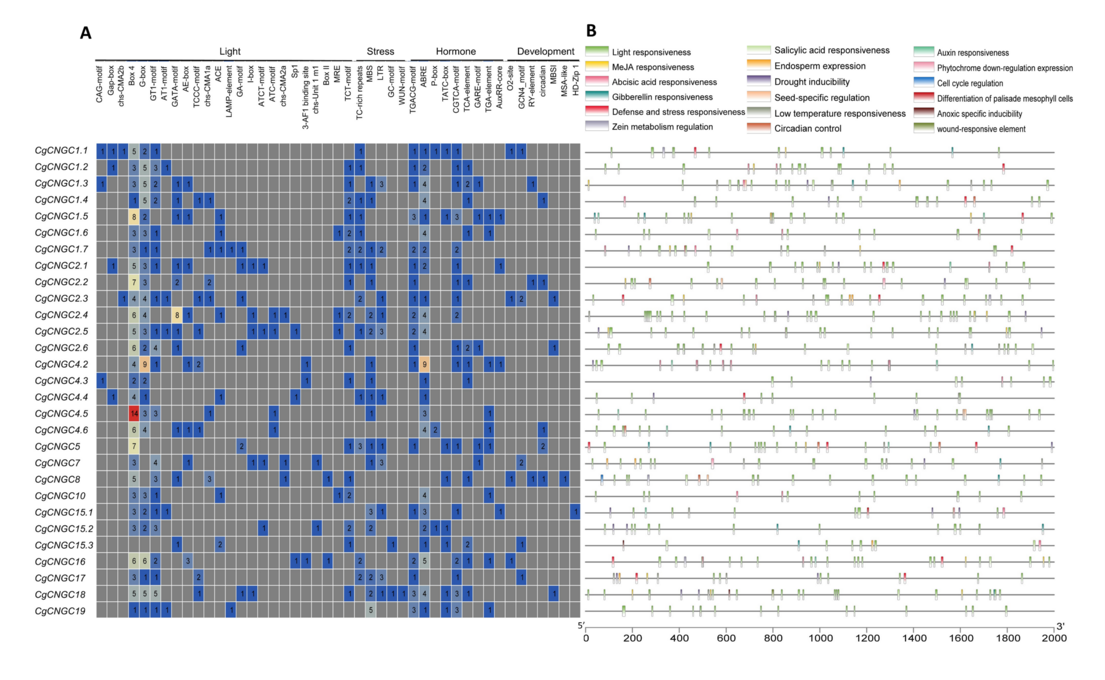


**Fig S15**: Cis-regulatory elements analysis of CgCNGCs.


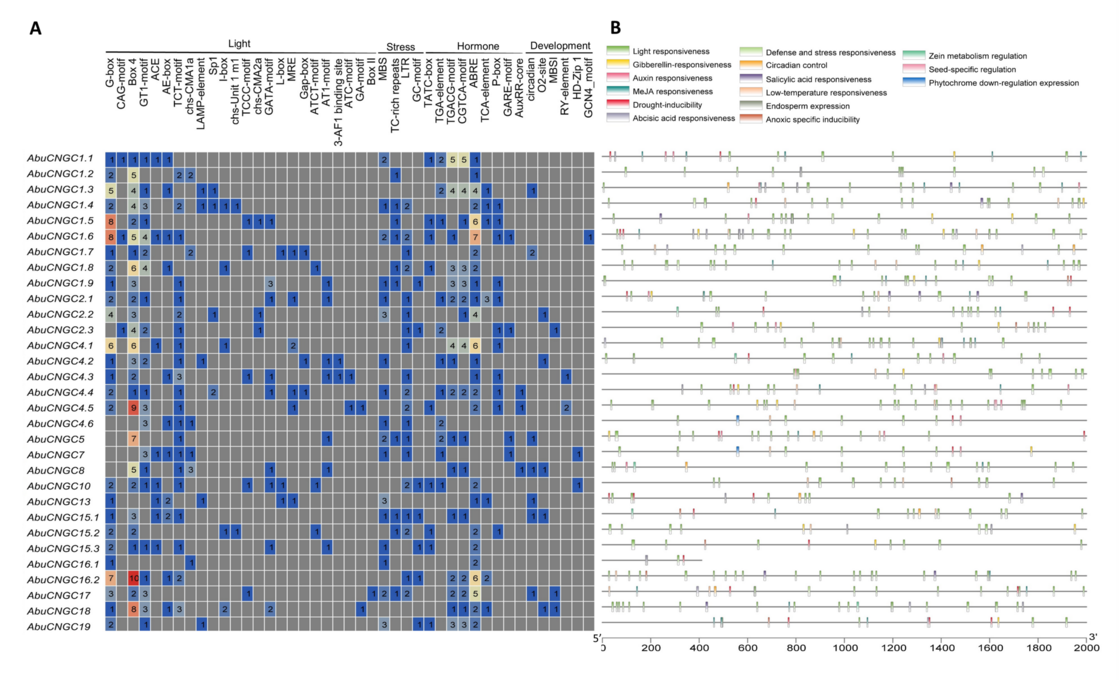


**Fig S16**: Cis-regulatory elements analysis of AbuCNGCs.


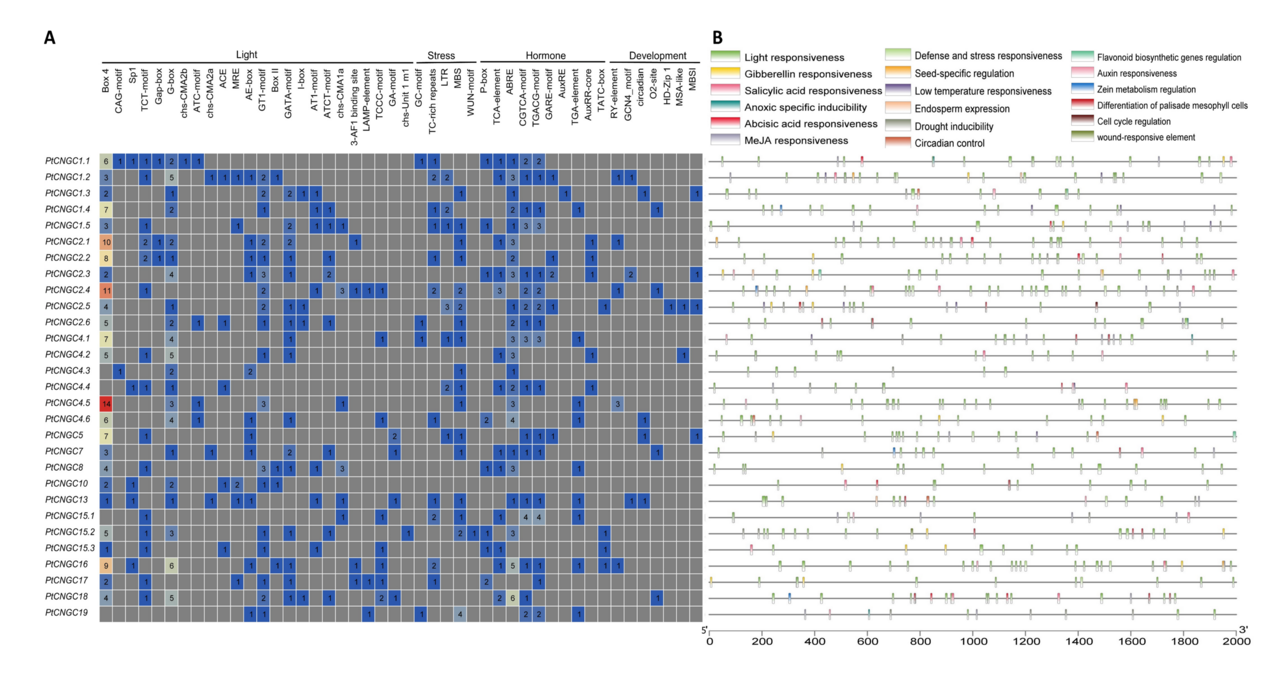


**Fig S17**: Cis-regulatory elements analysis of PtCNGCs.
