# Supplementary material for: Pangenome-wide analysis of cyclic nucleotide-gated channel (CNGC) gene family in citrus Spp. Revealed their intraspecies diversity and potential roles in abiotic stress tolerance
Source: Front Genet. 2022 Oct 11;13:1034921. doi: 10.3389/fgene.2022.1034921 (PMC9593079; doi:10.3389/fgene.2022.1034921)
Supplement: Supplementary file 1 [file DataSheet1.ZIP › Table S3. Gene duplication in CreCNGCs, GgCNGCs, AbuCNGCs, PtCNGCs..docx]

| **Gene 1** | | **Gene 2** | **Ka** | **Ks** | **Ka/Ks** | **Duplication Time (MYA)** | **Duplication type** |
| --- | --- | --- | --- | --- | --- | --- | --- |
| *CreCNGC2.2* | | *CreCNGC2.3* | 0.0484 | 0.0555 | 0.87207 | 4.230182927 | Tandem |
| *CreCNGC2.2* | | *CreCNGC2.4* | 0.4808 | 0.5035 | 0.95492 | 38.37652439 | Segmental |
| *CreCNGC2.3* | | *CreCNGC2.4* | 0.4851 | 0.5004 | 0.96942 | 38.1402439 | Segmental |
| *CreCNGC7* | | *CreCNGC8* | 0.1945 | 0.3116 | 0.6242 | 23.75 | Tandem |
| *CreCNGC15.1* | | *CreCNGC15.2* | 0.3179 | 0.5608 | 0.56687 | 42.74390244 | Tandem |
| *CgCNGC1.5* | | *CgCNGC1.6* | 0.2969 | 0.4523 | 0.65642 | 34.47408537 | Tandem |
| *CgCNGC2.1* | | *CgCNGC2.2* | 0.4486 | 0.6418 | 0.69897 | 48.91768293 | Tandem |
| *CgCNGC2.3* | | *CgCNGC2.4* | 0.0264 | 0.0225 | 1.17333 | 1.714939024 | Tandem |
| *CgCNGC2.3* | | *CgCNGC2.5* | 0.0398 | 0.075 | 0.53067 | 5.716463415 | Segmental |
| *CgCNGC2.3* | | *CgCNGC2.6* | 0.7355 | 0.908 | 0.81002 | 69.20731707 | Segmental |
| *CgCNGC2.4* | | *CgCNGC2.5* | 0.043 | 0.0544 | 0.79044 | 4.146341463 | Segmental |
| *CgCNGC2.4* | | *CgCNGC2.6* | 0.7226 | 0.87 | 0.83057 | 66.31097561 | Segmental |
| *CgCNGC2.5* | | *CgCNGC2.6* | 0.74 | 0.7772 | 0.95214 | 59.23780488 | Segmental |
| *CgCNGC7* | | *CgCNGC8* | 0.1745 | 0.2546 | 0.68539 | 19.4054878 | Tandem |
| *CgCNGC15.1* | | *CgCNGC15.2* | 0.2403 | 0.5122 | 0.46915 | 39.03963415 | Tandem |
| *AbuCNGC1.1* | | *AbuCNGC1.8* | 1.4362 | 1.8563 | 0.77369 | 141.4862805 | Tandem |
| *AbuCNGC1.1* | | *AbuCNGC10* | 0.6586 | 0.764 | 0.86204 | 58.23170732 | Tandem |
| *AbuCNGC1.2* | | *AbuCNGC1.7* | 0.6892 | 0.8521 | 0.80883 | 64.94664634 | Segmental |
| *AbuCNGC1.2* | | *AbuCNGC1.9* | 1.668 | 2.5584 | 0.65197 | 195 | Segmental |
| *AbuCNGC1.3* | | *AbuCNGC1.7* | 0.6892 | 0.8521 | 0.80883 | 64.94664634 | Segmental |
| *AbuCNGC1.3* | | *AbuCNGC1.9* | 1.668 | 2.5584 | 0.65197 | 195 | Segmental |
| *AbuCNGC1.8* | | *AbuCNGC1.9* | 0.7347 | 0.7476 | 0.98274 | 56.98170732 | Tandem |
| *AbuCNGC2.2* | | *AbuCNGC2.3* | 1.0532 | 1.7224 | 0.61147 | 131.2804878 | Segmental |
| *AbuCNGC7* | | *AbuCNGC8* | 0.1596 | 0.1491 | 1.07042 | 11.36432927 | Tandem |
| *PtCNGC1.4* | *PtCNGC1.5* | | 1.7976 | 1.4597 | 1.23149 | 111.257622 | Tandem |
| *PtCNGC2.1* | *PtCNGC2.3* | | 0.8124 | 1.2022 | 0.67576 | 91.63109756 | Tandem |
| *PtCNGC2.1* | *PtCNGC2.4* | | 0.8256 | 1.1011 | 0.7498 | 83.92530488 | Tandem |
| *PtCNGC2.2* | *PtCNGC2.3* | | 0.8133 | 1.1957 | 0.68019 | 91.13567073 | Segmental |
| *PtCNGC2.2* | *PtCNGC2.4* | | 0.8265 | 1.0956 | 0.75438 | 83.50609756 | Segmental |
| *PtCNGC2.3* | *PtCNGC2.4* | | 0.0295 | 0.0632 | 0.46677 | 4.817073171 | Tandem |
| *PtCNGC2.5* | *PtCNGC2.6* | | 1.4527 | 1.8386 | 0.79011 | 140.1371951 | Segmental |
| *PtCNGC5* | *PtCNGC8* | | 1.4191 | 1.5051 | 0.94286 | 114.7179878 | Segmental |
| *PtCNGC14* | *PtCNGC17* | | 0.739 | 1.0555 | 0.70014 | 80.44969512 | Tandem |
| *PtCNGC15.1* | *PtCNGC15.2* | | 0.343 | 0.5665 | 0.60547 | 43.17835366 | Tandem |
